# Saccharides-Based Polymers for Low Environmental Impact Adhesive Formulations

**DOI:** 10.1021/acsomega.5c11562

**Published:** 2026-03-18

**Authors:** Alice Cappitti, Emanuele Bianchini, Ursula Monaci, Daniele Martella, Benedetto Pizzo, Marco Frediani, Antonella Salvini

**Affiliations:** † Department of Chemistry “Ugo Schiff”, 9300 University of Florence, Via della Lastruccia 3-13, 50019 Sesto Fiorentino, Italy; ‡ CNR-IBE, Istituto per la BioEconomia (Institute of BioEconomy), via Madonna del Piano 10, 50019 Sesto Fiorentino, Italy

## Abstract

The growing demand for sustainable materials has driven the development of polymeric formulations containing biomonomers and/or bioadditives derived from renewable resources for high-performance applications. Among the various industrial sectors, the identification of more sustainable comonomers for classic adhesive formulations, such as vinyl and acrylic systems, is of particular relevance. In this context, the presence of hydroxyl functionalities is essential to promote post-cross-linking during joint formation, thereby ensuring suitable resistance to high thermohygrometric stresses. Saccharides are recognized as the most versatile building block to prepare biopolymers with different architectures; however, their industrial exploitation remains limited by the challenging preparation of polymerizable monomers, which could require complex multistep procedures. In this work, we describe the free radical copolymerization of allyl-functionalized saccharide monomers (allyl α,α′-trehalose, and allyl methyl glucopyranoside) with ethyl methacrylate and vinyl acetate to obtain copolymers suitable for nonstructural, post-cross-linkable adhesive applications. The selected saccharide monomers are characterized by a straightforward, protecting-group-free, one-step synthesis and have been previously employed for the preparation of low-molecular-weight polymers and oligomers, for example, in wood-protective applications. To extend their applicability to film-forming systems, such as adhesives, appropriate comonomers and molar ratios were chosen to achieve higher molecular weights, as required for effective adhesive performance. Indeed, the presence of a saccharide monomer allows the replacement of units with a high environmental impact, such as *N*-methylol acrylamide, commonly present in commercial adhesive formulations. Characterization by Nuclear Magnetic Resonance, Fourier transform infrared, differential scanning calorimetry, and size exclusion chromatography was performed to determine the composition, thermal properties, and molecular weight distribution. Adhesion tests on beech wood specimens have been performed both with and without an isocyanate cross-linker to demonstrate the potential of our saccharide-based copolymers as renewable components for adhesive systems with low environmental impact, providing an alternative to conventional formulations.

## Graphical abstract

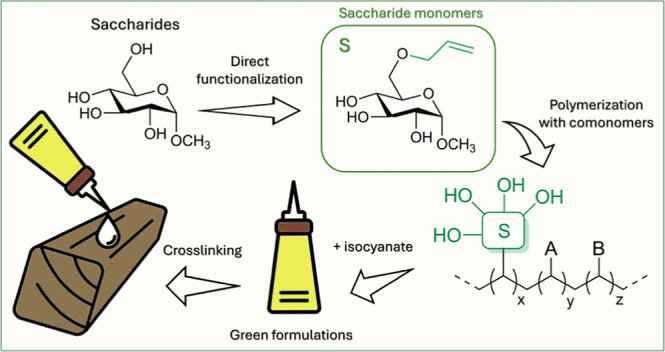

## 1 Introduction

The development of low environmental impact and more sustainable polymer formulations is a key objective in the industrial production of adhesives and other film-forming materials with binding functions. Increasingly stringent regulations on health and environmental protection require the improvement of widely used commercial formulations through replacement of toxic components. These needs are also in line with the environmental concerns associated with the extensive use of petroleum-derived polymers,^1^ and for this reason, increasing attention has been devoted to the development of sustainable and biocompatible materials. In particular, significant efforts have focused on biomonomers obtained from renewable resources,^2^ especially those originating from waste streams of industrial processes or from land management residues. These biobased monomers (“green monomers”) can be used for the synthesis of biopolymers or for copolymerization in already consolidated polymers, and when they are derived from saccharides, they hold significant appeal due to their affordability and widespread availability. Additionally, they are nontoxic to humans, exhibit minimal environmental impact, and offer interesting structural characteristics with the presence of multiple functionalities.^3^ Saccharides can be derived from lignocellulosic biomass using biorefinery processes: after separation of the main biomass components,^4,5^ cellulose and hemicellulose can be depolymerized into simple monosaccharides (glucose, xylose, mannose, and galactose).^6^ Glucose, the primary degradation product of cellulose, is the most extensively studied monosaccharide due to its high suitability for bioethanol production as well as for the synthesis of a broad range of value-added compounds such as 5-hydroxymethylfurfural (5-HMF) and levulinic acid.^7^ Also, methyl α-d-glucopyranoside can be obtained either through the direct methylation of glucose^8^ or via the hydrolysis and subsequent methylation of cellulose and d-cellobiose.^9^ It represents a valuable synthetic building block, primarily due to the protection of its anomeric position. Another noteworthy starting material is α,α′-trehalose, a nonreducing disaccharide. Although naturally abundant in a wide range of organisms, including algae, bacteria, fungi, insects, and certain plants,^10^ the large-scale industrial production of trehalose only became feasible following the development of an enzymatic synthesis process from starch in 1994. Due to the high stability of its glycosidic linkage, trehalose exhibits lower susceptibility to hydrolysis, rendering it more inert compared to other commonly used nonreducing sugar.^11^

Reactive monomers can be synthesized starting from these saccharide units by introduction of polymerizable groups, such as allyl or acrylate, on the hydroxyl groups, while the presence of residual hydroxyl groups maintains the reactive sites on the polymer chains obtained from their polymerization. A major bottleneck on their use is related to their complex multistep synthesis, including protecting and deprotecting steps, that generally limit interest in their possible industrial scalability. For instance, a series of monofunctional (meth)­acrylate or allyl derivatives have been described using glycosylation or transesterification reactions starting from acetylated saccharides, glucose, sucrose, and chitosan.^12–15^

Several alternatives, such as enzymatic^16,17^ or grafting^18,19^ methods, have been studied to eliminate the protection and deprotection steps which are not optimal for a possible industrial scale-up. Recently, Papacchini et al.^20^ reported the synthesis of allyl derivatives of methyl glucoside and trehalose via a nucleophilic substitution reaction, avoiding the use of protection and deprotection steps.

Among the polymerization techniques applied to saccharide monomers,^21^ free radical polymerization is one of the most widely employed synthetic approaches also in the industrial processes due to its simplicity and versatility.^22^ This method does not require extremely high monomer or solvent purity and can operate effectively under a broad range of reaction conditions with diverse monomer functionalities. Its extensive industrial application has also contributed to the availability of cost-effective initiators, making it a practical and economical choice for large-scale polymer synthesis. Numerous studies in the literature report the use of free radical polymerization for the synthesis of glycopolymers.^23–26^ Additionally, trehalose-based polymers^11^ have been prepared also via step-growth polymerization, and cross-linked materials have been reported by curing trehalose-containing polymers to form thermoset resins or hydrogels. The crucial contribution of saccharide-based monomers and their corresponding polymers to sustainability is well documented, with applications spanning from biomedical systems^11,27^ to the development of value-added products.

For example, allyl saccharides have been employed in the synthesis of vinyl acetate copolymers, as consolidants for degraded wood and paper materials.^28,29^ However, the reactivity of the allyl group acts as a chain terminator,^30,31^ hindering the conversion of the vinyl group and reducing the molecular weight of the copolymers, which can therefore be optimally used in consolidating treatments where the product must penetrate the micropores of the substrates. Conversely, this behavior represents a critical issue for the development of high molecular weight polymers that are required for other applications fields such as in the adhesives sector.

The adhesives properties of saccharide-based compounds, from natural one (starch and polysaccharide gums^32^) to synthetic cellulose derivatives,^33^ arise from high molecular weights and the presence of polar groups, enabling strong adhesion to polar substrates. In recent decades, saccharide derivatives have been also proposed as comonomers to enhance the properties of adhesion, improve biodegradability, and reduce toxicity of commercial polymeric formulations.^34^

A relevant example is the need to substitute harmful compounds like *N*-methylol acrylamide (NMA^35^), a widely used cross-linking agent that improves mechanical strength and moisture resistance but releases toxic formaldehyde.^36^ Despite their extensive use in the wood industry due to their excellent mechanical performance and durability, NMA-containing adhesives are increasingly subject to specific regulatory^37^ provisions due to health risks. In this context, the introduction of saccharide-based comonomers represents a promising strategy to provide hydroxyl-rich structures capable of promoting post-cross-linking reactions while avoiding the use of formaldehyde-releasing additives. The simultaneous presence of hydroxyl and polymerizable groups in saccharide-derived monomers may emulate the reactivity of *N*-methylol acrylamide by first promoting polyaddition copolymerization and in a later stage also cross-linking reactions with agents such as isocyanates, offering a potential way to reduce the toxicity of the final materials while equally improving water resistance.

In the present work, saccharide monomers were employed for the synthesis of polymers intended for use as adhesives in greener formulations, as described in Figure 1.

**Figure 1:**
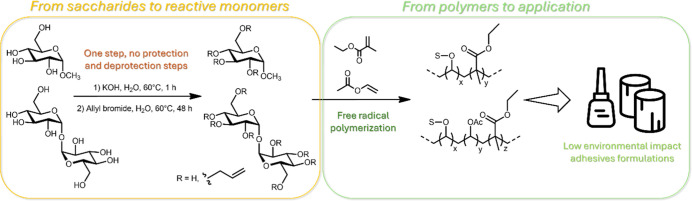
From monomers and polymers design to adhesives application.

A series of ethyl methacrylate and vinyl acetate copolymers were synthesized by introducing allylic derivatives of trehalose and methyl glucoside, in various molar ratios. The comonomers and their ratios were selected to modulate the reactivity of the final products and overcome the low molecular weights reported in the literature,^20^ with the aim of broadening their potential applications as adhesives or binding components in film-forming formulations. The mechanical performance of film-forming materials is well-known to depend on the physical and chemical properties of the polymer, including the molecular weight. Indeed, the selected copolymers were finally tested as wood adhesives in organic solvent-based formulations both in the presence and in the absence of isocyanates and then compared with the corresponding homopolymers of vinyl acetate and ethyl methacrylate synthesized under the same conditions. This analysis also shows the ability of the saccharide hydroxyl groups to participate in cross-linking reactions to improve the adhesion performances.

## 2 Experimental Session

### 2.1 Materials

α,α′-Trehalose, α-methyl-d-glucopyranoside, allyl bromide (99%), potassium hydroxide, hydrochloric acid 37%, sodium bicarbonate, potassium carbonate (≥99.0%), ethanol (96%), methanol (99.8%), vinyl acetate (>99%), ethyl methacrylate, and methyl ethyl ketone (≥99%) were purchased from Merck. Acetone (99.9%) and chloroform (>99%) were purchased from Carlo Erba. Azobis­(isobutyronitrile) was purchased from Fluka Co. Easaqua M502 was kindly supplied by Vencorex Chemicals.

### 2.2 Instruments

The ^1^H and ^13^C NMR spectra were recorded with a Varian Mercury plus 400 spectrophotometer or with a Varian INOVA spectrophotometer, both operating at a frequency of 339.921 MHz. The chemical shifts are reported in ppm and referred to TMS as the internal standard. Spectra elaboration was performed with the software MestReNova.

FT-IR spectra were recorded with a Shimadzu FT-IR spectrophotometer model IRAffinity-1S, connected to a computer with the lab Solution IR version 2.16 software, for data processing. All FT-IR analyses were set with the following parameters: spectral resolution of 4 cm^–1^; 32 scans for each spectrum, and scan range between 4000 and 400 cm^–1^.

Differential scanning calorimetry (DSC) measurements were performed using a TA Instruments Calorimeter Q-2000 DSC calorimeter (TA Instruments, Milan, Italy) in a nitrogen atmosphere (heating and cooling rate 10 °C/min). Thermogram processing was performed using the TA universal analysis software.

Molecular weight analyses were performed by size exclusion chromatography (SEC), using a system composed of a Shodex ERC-3215α degasser connected to a Waters 1525 binary HPLC pump, a Waters 1500 series heater set at 50 °C, a Wyatt miniDAWN TREOS detector, a Wyatt Viscostar-II and a Wyatt OPTILAB T-rEX detector, a Shodex GPC KF-802.5 column, and a Shodex GPC KF-803 column, using DMF as the eluent, maintaining a flow rate of 0.65 mL/min. A 5 mg/mL solution in DMF was prepared for each sample.

Adhesion tests were performed using an Instron mod. 5567 dynamometer with a 30 kN load cell and 0.5% accuracy. The dynamometer consists of a load cell, which measures the force applied to the specimen, and a displacement transducer. The specimen was mounted vertically, with the adherend without the hole clamped in the dynamometer, while the adherend with the hole was anchored to the device using a metal rod with a diameter 2 mm smaller than the hole to minimize stress during positioning. The distance between the dynamometer jigs (50 mm), the load bar speed (10 mm/min), the wood species (*Fagus sylvatica* L., i.e., beech), and the minimum detectable force (30 N) were kept constant for all tests. In this configuration, tangential stresses were applied to the bonding plane and gradually increased, until specimen failure occurred. Tangential tensile strength was then calculated as the ratio between the peak force and the bonding area (specified in Section 2.5.2). Stiffness was assessed as the slope of the force–displacement curve calculated automatically by machine software in the linear initial region.

### 2.3 Synthesis of Monomers

#### 2.3.1 Synthesis of Allyl α,α′-Trehalose (ATR)^20^

ATR was synthesized by modifying a previously reported procedure to enable process scale-up. In a Schlenk tube, an aqueous solution of KOH (42 mL, 2.10 M) was added under a nitrogen atmosphere to α,α-trehalose (11.1 mmol), and the mixture was heated at 60 °C for 1 h. After cooling to RT, allyl bromide (2.90 mL, 33.2 mmol) was added under a nitrogen atmosphere, and the mixture was allowed to react at 60 °C for 48 h under vigorous stirring. After cooling to RT, the pH was adjusted to neutrality using HCl (2 N), and finally, the solvent and the residual allyl bromide were distilled at reduced pressure. The solid residue was washed with ethanol to separate the product from the salts. The solvent was distilled at reduced pressure, and a white solid was obtained (4.35 g, 94% yield). The product was characterized by ^1^H NMR (see Figure S2, Supporting Information).

#### 2.3.2 Synthesis of Allyl Methyl d-Glucopyranoside (AMG)^20^

AMG was synthesized following a modified literature procedure, in which the reaction concentration was increased to improve the yield and facilitate subsequent scale-up. In a Sovirel tube, an aqueous solution of KOH (1.00 mL, 5.75 M) was added under a nitrogen atmosphere to methyl d-glucopyranoside (1.55 mmol), and the mixture was heated at 70 °C for 1 h. After cooling to room temperature, allyl bromide (0.42 mL, 4.85 mmol) was added under a nitrogen atmosphere, and the reaction mixture was allowed to react at 70 °C for 48 h under vigorous stirring. After the mixture was cooled to room temperature, the pH was adjusted to neutrality using HCl (2 N), and the solvent and the residual allyl bromide were distilled at reduced pressure. The resulting solid was washed with ethanol to separate the product from the salts. The alcoholic phase was distilled at reduced pressure, and a pale-yellow solid was obtained (341 mg, 91% yield). The product was characterized by ^1^H NMR (see Figure S3, Supporting Information).

### 2.4 Synthesis of Polymers

#### 2.4.1 Synthesis of Ethyl Methacrylate (*p*EMA) or Vinylacetate (*p*VAc) Homopolymers

Under a nitrogen atmosphere, degassed methanol (MeOH) and EMA or VAc were added to a Sovirel tube containing azobis­(isobutyronitrile) (AIBN) (Table 1). Then, the reaction mixture was allowed to react at 90 °C for 6 h under continuous stirring. After the mixture was cooled to room temperature, the solvent and the residual monomer were distilled at reduced pressure, and a white solid was obtained (*p*HEMA: 996 mg, yield 99.6%; *p*VAc: 982 mg, yield 98.2%). Homopolymers were characterized by ^1^H NMR, SEC, and DSC (results are reported in the Supporting Information).

**Table 1:** Summary of the Reagents Used in the Different Polymerizations

	EMA (mL)	VAc (mL)	ATR (mg)	AMG (mg)	AIBN (mg)	MeOH (mL)	H_2_O (mL)
*p*EMA	1.09	/	/	/	41.1	2.20	/
*p*VAc	/	1.07	/	/	54.4	1.74	0.77
CP1	0.68	/	200	/	18	2	/
CP2	0.90	/	100	/	37.2	2.80	/
CP3	1.50	/	100	/	57.2	4.60	/
CP4	0.81	/	/	50	33.3	2.51	/
CP5	1.08	/	/	50	45.5	3.44	/
CP6	1.31	/	/	50	54.5	4.12	/
TP1	0.03	0.22	100	/	13.1 + 10^a^	1.20	0.30
TP2	0.06	0.44	100	/	25.8 + 10^a^	1.68	0.42
TP3	0.03	0.20	/	50	11.8 + 10^a^	1.08	0.20
TP4	0.06	0.40	/	50	23.2 + 10^a^	1.51	0.38

a Added in two steps.

#### 2.4.2 Synthesis of EMA/ATR and EMA/AMG Copolymers (CP1–CP6, Table 1)

Under a nitrogen atmosphere, degassed methanol and EMA were added to a Sovirel tube containing ATR or AMG and AIBN (Table 1). Then, the reaction mixture was allowed to react at 90 °C for 6 h under continuous stirring. For CP1, after cooling to room temperature, the presence of a precipitate was observed and separated by centrifugation. For all products (CP1–CP6) after cooling the methanol solution to room temperature, the solvent and the residual ethyl methacrylate were distilled at reduced pressure, and the solid was washed with water. A white, water-insoluble solid was obtained. Copolymers were characterized by ^1^H NMR, SEC, and DSC (results are reported in the Supporting Information).

#### 2.4.3 Synthesis of ATR/VAc/EMA and AMG/VAc/EMA Terpolymers (TP1–TP4, Table 1)

Under a nitrogen atmosphere, degassed methanol, water, and VAc were added to a Sovirel tube containing ATR or AMG and AIBN (Table 1). The reaction mixture was allowed to react at 90 °C for 2 h under continuous stirring. After the mixture cooled to room temperature, EMA and more AIBN were added (Table 1). Then, the reaction mixture was allowed to react at 90 °C for 4 h under continuous stirring. After cooling the methanol solution to room temperature, the solvent and the residual EMA and VAc were distilled at reduced pressure, and the solid was washed by water. A pale-yellow water-insoluble solid was obtained and characterized by ^1^H NMR, SEC, and DSC (results are reported in the Supporting Information).

### 2.5 Applicative Tests

#### 2.5.1 Reactivity Tests with Isocyanates

Easaqua M502 (50% w/w with respect to polymer) was added to a 40% (w/w) solution of each polymer in methyl ethyl ketone (MEK). The samples were left to react for 10 days in an open container. Subsequently, 1 mL of MEK was added to each sample, and they were stirred overnight. The two phases were separated by centrifugation and dried by solvent evaporation at room temperature. The MEK-soluble fraction was weighed to determine the solubility of the sample and the percentage reduction in solubility compared to the initial product without the addition of isocyanates.

#### 2.5.2 Adhesion Tests

For each adhesive formulation, 7 beech wood specimens were prepared, consisting of two adherends (60 × 20 mm^2^). The bonding area (20 × 10 mm^2^) at the end of each adherend was defined using adhesive tape to confine the area of adhesive application (Figure S1, Supporting Information). To minimize the stress applied to the specimen when positioning it in the dynamometer jigs, a hole was made in one of the two wooden adherends. Polymer solutions were prepared in MEK, chosen for its ability to dissolve all tested polymers. Starting from the data about the reactivity with isocyanates, formulations were investigated both with and without the addition of isocyanates, with the aim of promoting cross-linking. Thus, for all six adhesives, two 40% (w/w) solutions in MEK were prepared: one containing only the adhesive and MEK and the other including the adhesive and the isocyanate cross-linker (Easaqua M502). The amount of adhesive solution was determined based on the standard practice^36^ and was set to 75 g/m^2^ of dry adhesive on each adherend. Considering that the bonding area was 200 mm^2^ and the adhesive concentration was 40% (w/w), the amount of solution spread on each adherend was 75 mg. This quantity was applied using a syringe, adjusting volumes based on the measured density of each solution (more details on solution preparation for adhesion tests are reported in the Supporting Information).

After the various solutions had been applied, the adherends were overlapped and kept in contact for a closed time of 10 min. They were then subjected to constant pressure for 2 h using a 35 kg weight. The specimens were then left for 3 days without compression to allow for solvent evaporation, evaluating whether a constant weight was reached. The specimens were subsequently stored in a thermostatic chamber at 21 °C and 60% relative humidity for 4 days prior to testing to complete the total 7 day conditioning period required by the standard UNI EN 205:2016.

## 3 Results and Discussion

### 3.1 Synthesis of Saccharide Monomers

Saccharide monomers were synthesized from α,α′-trehalose and methyl α-d-glucopyranoside optimizing a procedure previously described^20^ in view of a possible scale-up. Allyl α,α′-trehalose (ATR) was synthesized via nucleophilic substitution with allyl bromide (Figure 1), using a 3:1 molar ratio. The procedure was optimized to allow for scale-up to 4 g of starting material. The obtained substitution degree (DS) was estimated to be 1.0 by ^1^H NMR spectroscopy (Figure S2, Supporting Information), following the previously reported formula (eq S1, Supporting Information). Allyl methyl d-glucopyranoside (AMG) was obtained through a similar reaction with allyl bromide (Figure 1) (3:1 molar ratio), applying a higher reaction concentration (300 mg/mL) compared with the literature. The product exhibited a DS of 0.97, as determined by ^1^H NMR (Figure S3 and eq S2, Supporting Information).

The insertion of allyl groups into the saccharide structures enables their use as comonomers in radical copolymerization with vinyl and acrylic monomers, allowing the synthesis of biobased copolymers containing saccharide units. However, the reactivity of the selected monomers required the design of suitable copolymers or terpolymers with appropriate synthetic procedures to overcome the chain terminator effect described above for vinyl derivatives.

### 3.2 Synthesis and Characterization of Acrylic Copolymers

The synthesis of copolymers with ATR or AMG was carried out using ethyl methacrylate as a comonomer at different starting molar ratios (Table 2). The presence of a highly reactive comonomer such as EMA was exploited to enhance the reactivity of the allylic saccharide monomer. Compositions with a higher saccharide monomer content were selected to facilitate proper characterization of the final products, while those with lower saccharide content were chosen to better reflect conditions of industrial relevance for use in adhesive formulations.

**Table 2:** Synthesis of Copolymers between ATR or AMG and EMA

	starting molar ratio	copolymer yield (%)^a^	EMA conversion (%)^b^	ATR/AMG conversion (%)^c^	final unit ratio by weight^d^	final unit ratio by ^1^H NMR^e^
CP 1 (EMA/ATR)	10	70.7	98.8	34.7	27.9	28
CP 2 (EMA/ATR)	30	92.6	98.0	53.0	54.2	55
CP 3 (EMA/ATR)	50	94.4	98.7	63.8	78.7	80
CP 4 (EMA/AMG)	30	90.8	98.1	75.0	38.3	36.6
CP 5 (EMA/AMG)	40	89.9	98.2	74.4	50.9	55.5
CP 6 (EMA/AMG)	50	95.9	98.7	80.6	64.2	/

a Calculated based on the weight of the corresponding fraction compared to the theoretical weight.

b Calculated considering the volatility of unreacted EMA and based on the difference between the weight of the residue at the end of the work up and the theoretical one.

c Calculated based on the quantity of unreacted monomer in the water-soluble extraction.

d Calculated considering the monomers conversion.

e Calculated based on the integrals of the monomeric units, as described in the text.

The reaction was conducted as a radical polymerization in organic solvent (MeOH), using AIBN as the radical initiator, following the schemes reported in Figure 2.

**Figure 2:**
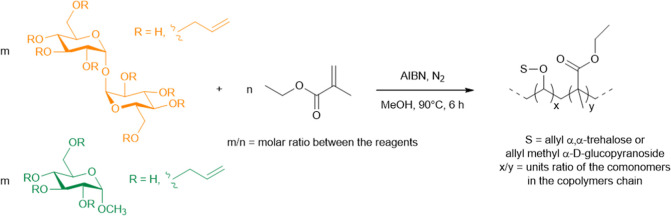
Synthesis schemes of EMA/ATR and EMA/AMG copolymers.

In the case of CP1, at the end of the reaction, two separate phases were found: a white solid and a methanol soluble fraction. The former showed only characteristic signals corresponding to the EMA homopolymer in the NMR spectrum. Conversely, the methanol-soluble fraction exhibited signals indicative of both monomers for the EMA/ATR copolymer and the residual unreacted ATR monomer, as evidenced by the presence of the allyl group. To isolate and quantify the unreacted saccharide monomer, the residue obtained after evaporation of the methanol and the unreacted acrylic monomer was subjected to successive water washing. Indeed, the resulting water-soluble extract displayed only signals corresponding to the ATR monomer, while the ^1^H NMR spectrum of the water-insoluble fraction confirms the presence of the EMA/ATR copolymer (Figure 3a) for the presence of the signals attributable to both the acrylate and saccharide units. In particular, between 0.89 and 1.06 ppm, the signals due to –CH_2_–C­(C**H**_**3**_) are present, together with 1.29 ppm (–COO–CH_2_–C**H**_**3**_), 1.91, 1.96 ppm (–C**H**_**2**_–C–(CH_3_)–), and at 4.06 the signal attributable to –COO–C**H**_**2**_–CH_3_, while for the saccharide unit, the signals relating to the 10 H of trehalose are present between 3.44 and 3.95 ppm (**H**_**3**_ **–H**_**6**_ and **H**_**3**_ **′–H**_**6**_ **′**).

**Figure 3:**
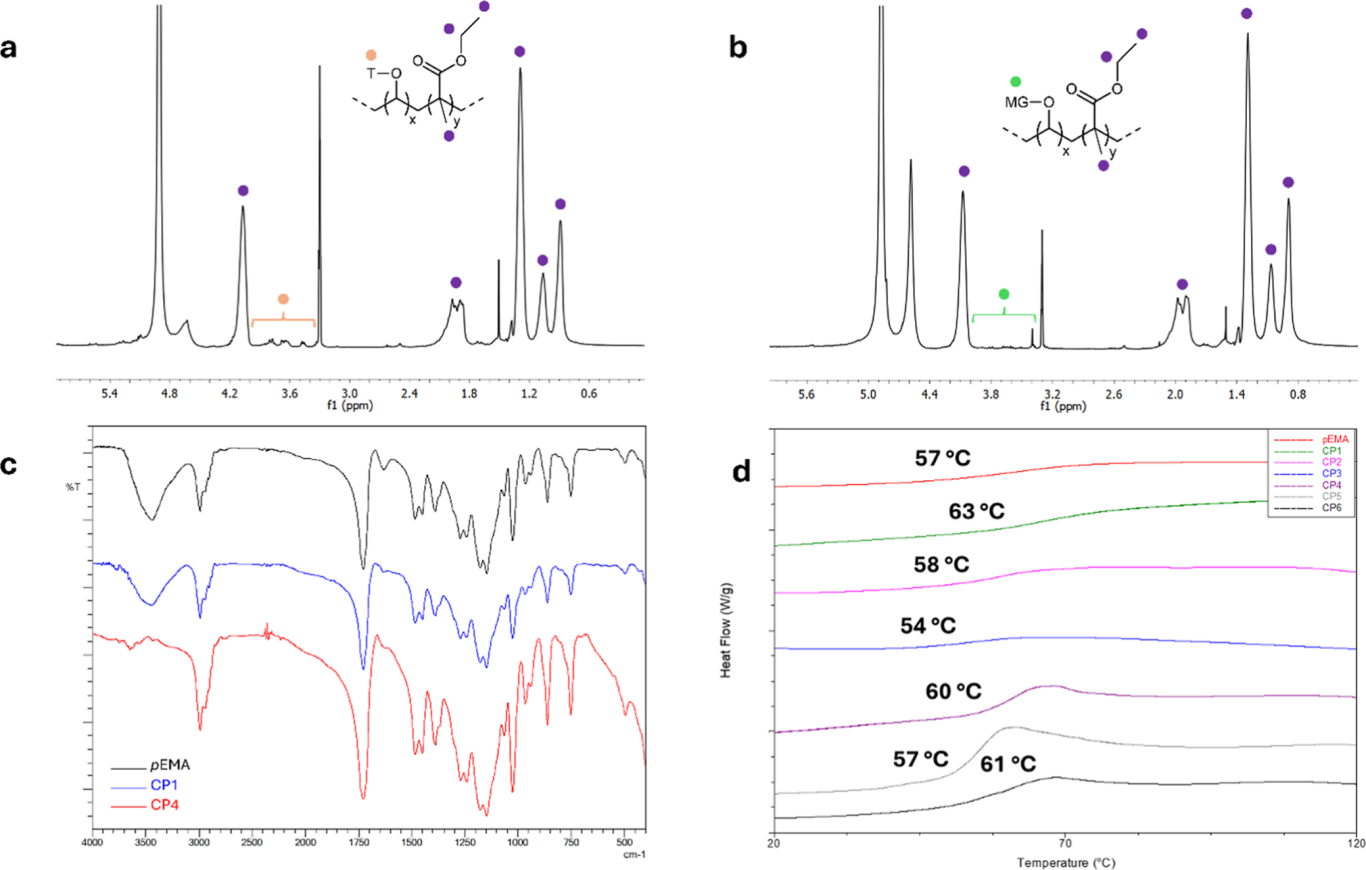
Copolymers characterizations: (a) ^1^H NMR spectrum (CD_3_OD) of water-insoluble fraction of CP1; (b) ^1^H NMR spectrum (CD_3_OD) of water-insoluble fraction of CP4; (c) FT-IR spectra of *p*EMA, CP1, and CP4; and (d) DSC analysis.

For the other copolymers, only a soluble phase was present at the end of the reaction. After the evaporation of the volatile components, ^1^H NMR analysis revealed signals corresponding to the copolymer together with the presence of unreacted ATR or AMG monomers. Consequently, aqueous washing was carried out to remove the unreacted saccharide monomers and obtain the desired copolymer products in the water-insoluble fractions (Figure 3b shows the ^1^H NMR spectrum of CP4 for example, others are reported in the Supporting Information, Figures S4–S7).

In all cases, the conversion of monomers and the final ratio between the starting monomers were determined by gravimetric analysis. In fact, the quantity of unreacted ATR or AMG was evaluated from the weight of the water-soluble fraction which contains the monomer extracted from the reaction mixture, while EMA conversion was determined by comparing the theoretical mass with the final dry residue, as unreacted ATR or AMG remains in the solid phase and the unreacted EMA is evaporated in the work up. Conversion data (Table 2) confirm the high reactivity of EMA, with nearly complete conversion in all cases. ATR shows lower conversion but improves slightly at higher EMA/ATR ratios, while AMG achieves higher and more stable conversions, largely unaffected by feed composition. By considering the conversions of both monomers, the molar ratio of the incorporated units can also be determined.

When possible, the final unit ratio was also calculated by ^1^H NMR, as follows (eqs 1 and 2)­

1 $$F\text{or}\;EMA/ATR\;copolymers:\frac{EMA}{ATR}=\frac{A/6}{B/10}$$

where **A** is the integral of the signal between 0.89 and 1.29 which corresponds to the 6 H of the ethyl methacrylate unit (–CH_2_–C­(C**H**_**3**_)– and –COO–CH_2_–C**H**_**3**_); **A/6** corresponds to the 1 H of the EMA unit; **B** is the integral of the signal between 3.44 and 3.95 ppm which corresponds to 10 H of the saccharide unit (**H**_**3**_ **–H**_**6**_ and **H**_**3**_ **′–H**_**6**_ **′**); and **B/10** corresponds to the 1 H of the saccharide unit.

2 $$\text{For}\;EMA/AMG\;copolymers:\frac{EMA}{AMG}=\frac{A/6}{B/9}$$

where **A** is the integral of the signal between 0.90 and 1.30 which corresponds to the 6 H of the ethyl methacrylate unit (–CH_2_–C­(C**H**_**3**_)– and –COO–CH_2_–C**H**_**3**_); **A/6** corresponds to the 1 H of the EMA unit; **B** is the integral of the signal between 3.34 and 3.96 ppm which corresponds to the 9 H of the saccharide unit (**H**_**3**_ **–H**_**6**_ and **H**_**3**_ **′–H**_**6**_ **′**); and **B/9** corresponds to the 1 H of the saccharide unit.

Data obtained by NMR analysis (Table 2) also confirmed the reliability of the gravimetric data. In the case of AMG/EMA 1/50, due to the lower molecular weight compared to ATR and the low AMG content, it was not possible to determine the ratio by ^1^H NMR.

FT-IR analyses were also performed on *p*EMA and copolymers (examples are reported in Figure 3c). The presence of the monomeric unit derived from EMA is confirmed by the intense band at 1728 cm^–1^ corresponding to C=O stretching. Additionally, C–O–C stretching from the EMA unit is observed between 1254 and 966 cm^–1^. However, due to the high ratio between the two monomer units, the saccharide bands (–C–OH stretching) are not distinguishable as they are overlapped by the EMA bands, influencing only the relative intensities of the bands.

Copolymers were finally characterized using SEC and DSC (Table 3 and Figure 3d) and the data were compared with an EMA homopolymer (*p*EMA) synthesized under the same reaction conditions.

**Table 3:** *T*_g_ and Molecular Weight Values of Copolymers and *p*EMA

	*T*_g_ (°C)^a^	*M*_n_ (g/mol)^b^	*M*_w_ (g/mol)^c^	*D̵* ^d^
*p*EMA	57	24,810	38,020	1.53
CP1 (EMA/ATR = 27.9)	63	20,800	34,470	1.65
CP2 (EMA/ATR = 54.2)	58	21,640	34,530	1.59
CP3 (EMA/ATR = 78.7)	54^a^	23,340	46,870	2.01
CP4 (EMA/AMG = 38.3)	60	44,520	66,050	1.30
CP5 (EMA/AMG = 50.9)	57	46,330	72,220	1.56
CP6 (EMA/AMG = 64.2)	61	32,770	40,800	1.24

a *T*_g_ refers to the 1st cycle for all samples, except for CP3, which refers to the 2nd cycle.

b Number-average molecular weight.

c Weight-average molecular weight.

d Dispersity index.

The differences observed in the results reported in Table 3 can be attributed to the chemical structure of the saccharide monomers, particularly to the number of hydroxyl groups and the steric hindrance associated with the saccharide units, which influence the properties of the resulting copolymers.

Regarding the glass transition temperature (*T*_g_), the homopolymer obtained with the reaction conditions selected in our procedure exhibited lower *T*_g_ than the commercial values (63–65 °C), confirming that the synthetic method has a measurable effect on the final properties. The presence of the saccharide comonomer does not appear to significantly influence the *T*_g_. For copolymers containing ATR, slight variations in *T*_g_ values are generally consistent with the comonomer unit ratios. CP1 exhibits a higher *T*_g_ compared with the EMA homopolymer. The *T*_g_ value progressively decreases with the reduction of sugar content, approaching that of pure *p*EMA. For CP3, a slightly lower value is observed than that of the homopolymer. It is important to note that in this case, *T*_g_ was observed during the second heating cycle. In the first cycle, an endothermic peak was detected at approximately the same temperature, overlapping with the *T*_g_ signal (see Figure S8, Supporting Information). For copolymers containing AMG (CP4, CP5, and CP6), the *T*_g_ value is equal to or slightly higher than that of the homopolymer, but a trend strictly correlated to the ratio of the comonomers is not observed. This irregularity may be attributed to the low AMG content, the synthetic approach, and sensitivity even to minimal variations in initiator mass, which can lead to differences in molecular weight and, consequently, in *T*_g_.

The molecular weights obtained are higher than those reported in the literature for copolymers with vinyl acetate,^20^ confirming the effectiveness of the strategy of introducing a more reactive monomer such as EMA to increase molecular weights. In these systems, the effect of the saccharide unit is mainly reflected in the molecular weight. SEC results indicate that the presence of ATR leads to a small reduction in molecular weight compared with EMA homopolymers. This effect is particularly evident in CP1, which exhibited the lowest molecular weight among the ATR-containing copolymers. As the EMA/ATR monomer ratio increases, the molecular weight correspondingly rises, approaching that of pure *p*EMA. In contrast, the incorporation of AMG results in a significant increase in molecular weight, with CP5 nearly doubling the molecular weight relative to *p*EMA. This behavior can be reasonably ascribed to the lower steric hindrance of the AMG moiety, which may facilitate chain growth during polymerization and promote the formation of larger polymer chains. CP6 showed a smaller increase in molecular weight; it exhibited a lower dispersity (*D̵*), indicating a more uniform polymer chain length distribution. Overall, the *D̵* values observed across all copolymers are consistent with the characteristics of conventional free radical polymerization, which typically yields dispersivity indices in the range 1.50 to 2.00.

### 3.3 Synthesis and Characterization of the Terpolymers

The synthesis of terpolymers with ATR and AMG was carried out by using VAC and EMA as comonomers at different starting molar ratios (Table 4). The addition of EMA, in a smaller quantity than VAc, was designed to try to overcome the reactivity limits obtained in the past^20^ with only VAc by increasing both the conversion and the MW. Compositions with a higher saccharide monomer content were selected to facilitate proper characterization of the final products, while those with lower saccharide content were chosen to better reflect conditions of industrial relevance for the use in adhesive formulations.

**Table 4:** Synthesis of Terpolymers between ATR or AMG, VAc and EMA

	starting molar ratio	final units ratio by ^1^H NMR^a^	terpolymer yield (%)^b^	EMA conversion (%)^c^	ATR/AMG conversion (%)^d^	VAc conversion (%)^e^
TP1 (ATR/VAc/EMA)	1/10/1	1/10.6/2.8	65.1	>98	54.9	57.9
TP2 (ATR/VAc/EMA)	1/20/2	1/22.2/3.4	83.5	>98	55.3	86.2
TP3 (AMG/VAc/EMA)	1/10/1	1/10.6/3.7	57.0	>98	44.0	61.5
TP4 (AMG/VAc/EMA)	1/20/2	1/17.3/3.7	72.3	>98	73.6	67.9

a Calculated based on the integrals of the monomeric units, as described in the text.

b Calculated based on the weight of the corresponding fraction compared to the theoretical weight.

c Assumed considering its reactivity in these conditions.

d Calculated based on the unit ratio by NMR and the number of repeating units.

e Calculated assuming unreacted allyl saccharide remains in the crude product and EMA conversion is >98%. From the crude product weight, the unreacted VAc was determined, and conversion was expressed as the ratio of converted to initial VAc.

The reaction scheme of the preparation of terpolymers is reported in Figure 4. A MeOH/H_2_O solvent mixture was selected as a solvent medium due to its ability to promote polymer conversion while accommodating the different solubilities of the comonomers. The synthesis was conducted in two steps to account for the different reactivities of the comonomers. In the first step, only saccharide monomers, VAc, and AIBN were allowed to react. Subsequently, additional AIBN and EMA were added to complete the reaction.

**Figure 4:**
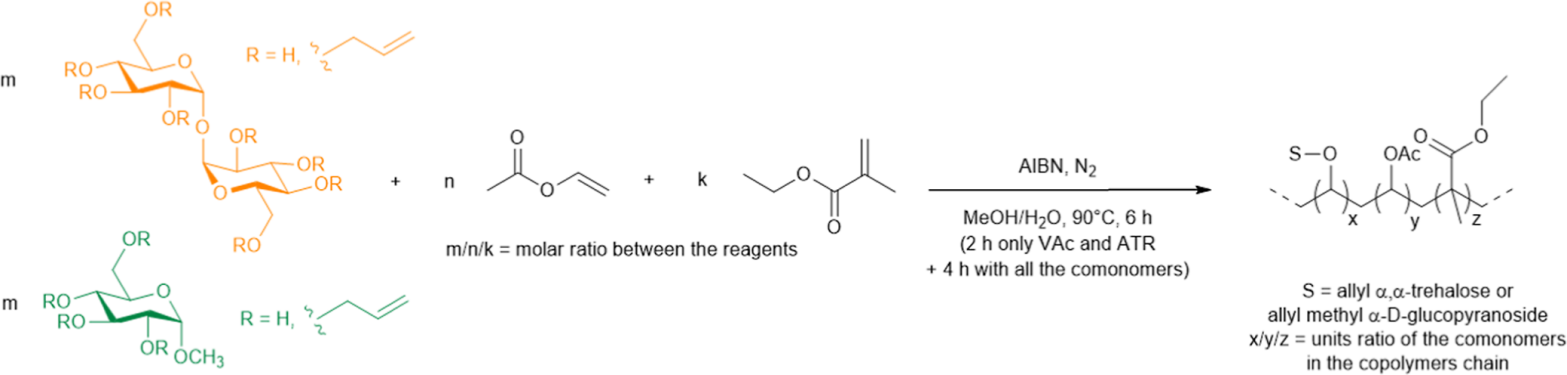
Synthesis schemes of ATR/VAc/EMA and AMG/VAc/EMA terpolymers.

All of the reactions produced homogeneous solutions, and the solid products were isolated by evaporating the solvent and other volatile components. The synthesis of terpolymers was confirmed by ^1^H NMR spectroscopy, with the presence signals corresponding to all three repeating units observed. The signals of unreacted allyl groups of the saccharide monomer were also observed, indicating a not complete conversion. Therefore, to remove any unreacted saccharide monomer, successive water washings were carried out. In this case, the ^1^H NMR spectra of the water-soluble fractions revealed the presence of signals attributed to unreacted ATR or AMG but also the VAc/allylsaccharide copolymer chains with higher saccharide monomer content, which makes them water-soluble (see the Supporting Information, Figures S9 and S10 show TP1 and TP3 as examples). The water-soluble fraction corresponded for all of the syntheses to an amount lower than 12% of the total final residue.

The water-insoluble fractions contained only the desired terpolymers products (Figure 5a,b shows the ^1^H NMR spectra of TP1 and TP3 for example, others are reported in the Supporting Information, Figures S11 and S12). Signals corresponding to the acrylate unit in the copolymer can be observed: between 0.90 and 1.07 ppm, signals attributable to –CH_2_–C­(C**H**_**3**_)–; at 1.27 ppm, the signal corresponding to –COO–CH_2_–C**H**_**3**_; and at 4.07 ppm, the signal attributable to –COO–C**H**_**2**_–CH_3_. The signals for vinyl acetate appear at 1.84 ppm (CH_3_–COO–CH–C**H**_**2**_–), 2.01 ppm (C**H**_**3**_–COO–CH–CH_2_–), and 4.98 ppm (CH_3_–COO–C**H**–CH_2_–). In TP1 and TP2, signals between 3.48 and 3.91 ppm correspond to the 10 hydrogen atoms of trehalose. In TP3 and TP4, signals between 3.34 and 3.96 ppm correspond to the 6 hydrogen atoms of AMG, partially overlapping to signal at 3.40 ppm attributable to –O–C**H**_**3**_. The signal at 4.66 ppm related to the anomeric proton is also present.

**Figure 5:**
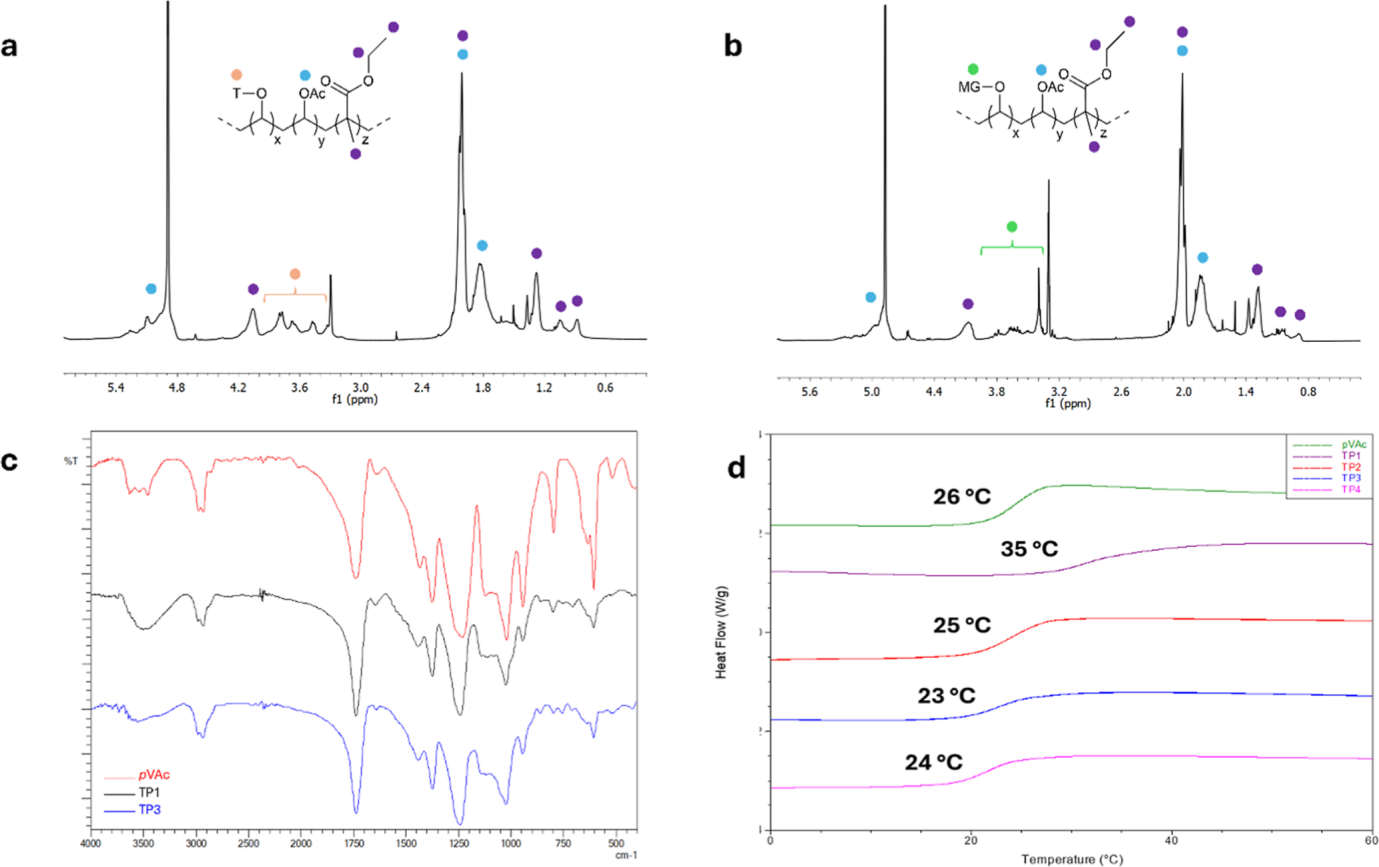
Terpolymers characterizations: (a) ^1^H NMR spectrum (CD_3_OD) of water-insoluble fraction of TP1; (b) ^1^H NMR spectrum (CD_3_OD) of water-insoluble fraction of TP3; (c) FT-IR spectra of *p*VAc, TP1, and TP3; and (d) DSC analysis (the reported values correspond to the *T*_g_, evaluated as the midpoint of the transition).

In all cases, the final ratio between the different monomer units was determined by ^1^H NMR (Table 4) with the following formula (eqs 3 and 4):

3 $$F\text{or}\;ATR/VAc/EMA\;terpolymers:ATR=\frac{A}{10},VAc=\frac{\left[B-\left(\frac{C}{6}\times 2\right)\right]}{5},EMA=\frac{C}{6}$$

where **A** is the integral of the signal between 3.45 and 3.95 ppm, corresponding to the 10 H of the ATR; **A/10** corresponds to the 1 H of the saccharide unit; **B** is the integral of the signal between 1.73 and 2.11 ppm, corresponding to the 5 H of the vinyl acetate; **[B – (C/6 × 2)]/5** corresponds to the 1 H of the VAc unit; (C/6 × 2) to eliminate the signal of –C**H**_**2**_–C­(CH_3_)– of EMA which is overlapped; **C** is the integral of the signal between 0.88 and 1.33 ppm, corresponding to the 6 H of the ethyl methacrylate; and **C/6** corresponds to the 1 H of the EMA unit.

4 $$\text{For}\;AMG/VAc/EMA\;terpolymers:AMG=\frac{A}{9},VAc=\frac{\left[B-\left(\frac{C}{6}\times 2\right)\right]}{5},EMA=\frac{C}{6}$$

where **A** is the integral of the signal between 3.34 and 3.96 ppm, corresponding to the 9 H of the AMG; **A/10** corresponds to the 1 H of the saccharide unit; **B** is the integral of the signal between 1.56 and 2.13 ppm, corresponding to the 5 H of the vinyl acetate; **[B – (C/6 × 2)]/5** corresponds to the 1 H of the VAc unit; (C/6 × 2) to eliminate the contribution of the signal of –C**H**_**2**_–C­(CH_3_)– of EMA which is overlapped; **C** is the integral of the signal between 0.76 and 1.42 ppm, corresponding to the 6 H of the ethyl methacrylate; and **C/6** corresponds to the 1 H of the EMA unit.

Based on the final unit ratios and the gravimetric analysis of the separated fractions, it is possible to estimate the incorporation of saccharide monomers into the terpolymer and consequently in the water-soluble extract (see Figures S13 and Table S1, Supporting Information). Finally, evaluating the variation in the degree of substitution within the water-soluble extract, both the percentage of unreacted allylsaccharides and the amount copolymerized with VAc can be determined (Figure S13 and Table S1, Supporting Information). Vac conversion was estimated by assuming that the unreacted allyl saccharide remains in the crude product, while EMA under these conditions shows a conversion higher than 98%. Therefore, by calculating the difference between the weight of the crude product and the total weight of the reagents and considering a EMA conversion of 98%, the amount of unreacted VAc, and consequently the amount of converted VAc, was determined. VAc conversion was then evaluated as the ratio between converted VAc and the initial VAc (Table 4). Considering the conversion data, EMA consistently exhibits a nearly complete conversion. ATR conversion is only slightly influenced by the feed composition. In contrast, AMG conversion is more dependent on the monomer ratio, with improved incorporation when its relative content is reduced. VAc shows variable behavior, with conversion influenced by both the comonomer type and the feed ratio, tending to be higher in ATR-based systems and moderate in those containing AMG.

FT-IR analyses were also performed on *p*VAc and terpolymers (examples are reported in Figure 5c). In the terpolymer spectra, the C=O stretching vibration is clearly observed at 1737 cm^–1^, while the C–O stretching band appears at 1242 cm^–1^. The –C–OH and C–O–C stretching vibrations are located in the 1120–1024 cm^–1^ range, with overlapping signals attributable to all present monomeric units derived from the saccharide, VAc, and EMA. When compared to the *p*VAc homopolymer spectrum, it is evident that the dominant *p*VAc signals overshadow the peaks of the other comonomers. The low amounts of EMA and ATR or AMG in the terpolymers make FT-IR characterization more challenging, as they only partially influence peak intensities without allowing a clear distinction of the ATR/AMG and EMA content relative to VAc across the different terpolymers.

Terpolymers were finally characterized using SEC and DSC (Table 5 and Figure 5d) and compared with a VAc homopolymer (*p*VAc) synthesized under the same reaction conditions.

**Table 5:** *T*_g_ and Molecular Weight Values of Terpolymers and *p*VAc

	*T*_g_ (°C)^a^	*M*_n_ (g/mol)^b^	*M*_w_ (g/mol)^c^	*D̵* ^d^
*p*VAc	26	41,730	73,910	1.77
TP1 (ATR/VAc/EMA = 1/10.6/2.8)	35	22,030	73,790	3.35
TP2 (ATR/VAc/EMA = 1/22.2/3.4)	25	39,710	69,810	1.76
TP3 (AMG/VAc/EMA = 1/10.6/3.7)	23	33,500	51,270	1.53
TP4 (AMG/VAc/EMA = 1/17.3/3.7)	24	47,490	84,380	1.78

a *T*_g_ refers to the 1st cycle for all samples.

b Number-average molecular weight.

c Weight-average molecular weight.

d Dispersity index.

The differences observed in the results reported in Table 5 can be attributed to the chemical structure of the saccharide monomers, particularly to the number of hydroxyl groups and the steric hindrance associated with the saccharide units, which influence the properties of the resulting terpolymers.

Also in this case, the VAc homopolymer exhibited a *T*_g_ lower than the literature (30 °C)^39^ and commercial values (32 °C). The presence of comonomers modifies the *T*_g_ with respect to the homopolymer, with a final result deriving from the different concomitant actions that makes it difficult to identify a regular trend. For terpolymers containing ATR, higher amounts of the saccharide monomer result in an increase in *T*_g_, as observed for TP1. However, with increasing VAc content, as in TP2, a decrease in *T*_g_ is noted. In contrast, terpolymers incorporating AMG exhibit *T*_g_ values that are slightly lower than those of the homopolymer in both the cases. This behavior may be attributed to the different molecular structures of the two saccharides: methylglucoside may act as a plasticizer, leading to a decrease in *T*_g_ through increasing chain mobility; conversely, trehalose is thought to promote a more rigid hydrogen-bonding network, which could limit chain movement and result in a higher *T*_g_.

Also in this case, the molecular weights obtained are higher than those reported in the literature,^20^ confirming the success of the strategy of introducing small amounts of EMA to achieve higher-molecular-weight terpolymers. Furthermore, the presence of ATR slightly reduces the molecular weight, as observed in TP1, whereas reducing the ATR content (TP2) restores values comparable to those of *p*VAc. Similarly, in the terpolymers containing AMG, the reduction of the saccharide content leads to an increase in the molecular weight, with TP4 even exceeding that of the *p*VAc homopolymer. As discussed for copolymers, the lower steric hindrance of the AMG moiety may facilitate chain growth during polymerization and promote the formation of larger polymer chains.

### 3.4 Application Tests: Reactivity with Isocyanates and Wood Adhesion

A reactivity study was carried out to assess the actual involvement of the saccharide moiety of the copolymers in cross-linking reactions. In polyisocyanate-based systems, polyurethane formation with hydroxyl groups is the main reaction, but concomitant polyurea cross-linking also contributes to performance.^40^ In water-based formulations, even trace amounts of water lead to isocyanate hydrolysis and subsequent polyurea formation, as the amines generated exhibit higher nucleophilicity than the hydroxyl groups and preferentially react with isocyanates. Both processes are known to occur in isocyanate-based systems and can contribute to cross-linking, thereby affecting mechanical properties and solubility. However, a more pronounced decrease in solubility can be observed for polymers containing hydroxyl-functional monomers, indicating their active involvement in the cross-linking process with the formation of polyurethane bonds.

For this study (Figure 6), the commercial oligomeric isocyanate Easaqua M502 was chosen as the product used in the formulation of commercial adhesives. Polymers, in MEK solution, were mixed with 50% w/w Easaqua M502, spread into films, and cured at room temperature for 10 days. Solvent extraction was then used to separate soluble and insoluble fractions, and gravimetric analysis assessed the reduction in solubility with respect to the corresponding copolymer without isocyanates as an indirect measure of cross-linking (Figure 6 and Table 6). Results were compared to similarly treated *p*VAc and *p*EMA homopolymers.

**Figure 6:**
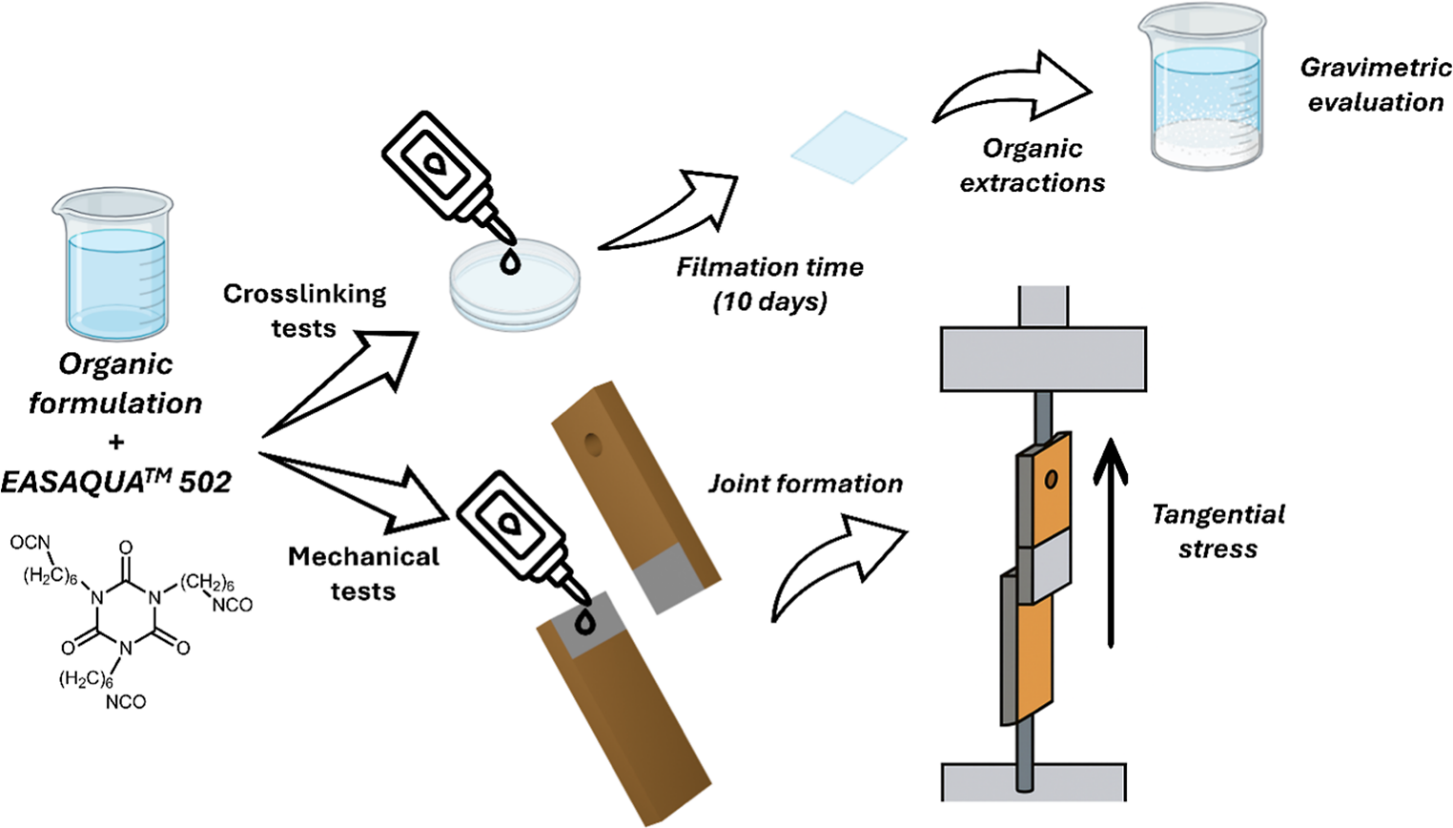
Applicative studies setup: formulations preparation; reactivity test with isocyanates and mechanical tests on wood specimens.

**Table 6:** Reactivity Tests with Isocyanate^a^

	solubility reduction (%) after 10 days
*p*EMA	40.2 ± 1.3
CP1	49.9 ± 1.0
CP2	47.6 ± 1.2
CP3	44.4 ± 0.5
CP4	45.8 ± 0.9
CP5	44.7 ± 0.8
CP6	42.8 ± 0.7
*p*VAc	46 ± 0.9
TP1	80.4 ± 1.9
TP2	74.9 ± 2.3
TP4	59.1 ± 1.5

a Reported data correspond to three independent replicates.

As expected, in saccharide-containing polymers, hydroxyl groups provide an additional cross-linking pathway via polyurethane formation, further decreasing solubility. The more pronounced reduction observed in all hydroxyl-functional polymers compared to homopolymers therefore supports their active role in the cross-linking process.

For EMA-based copolymers, the observed slightly pronounced differences are correlated with the saccharide monomer content and follow the trend of the hydroxyl content. These values are influenced by both the type and the amount of saccharide incorporated. Notably, CP4 exhibited the greatest reduction, which can be attributed to the presence of trehalose and the highest overall saccharide content. In contrast, CP6 and *p*EMA displayed nearly identical behavior. This is likely due to the high hydrophobicity of EMA units, which may hinder the accessibility or reactivity of saccharide hydroxyl groups. Additionally, the use of AMG, with fewer hydroxyl groups than ATR, combined with a high EMA/AMG ratio, results in a lower overall hydroxyl content, thereby limiting the extent of cross-linking. Terpolymers show a greater solubility reduction than their corresponding homopolymers, with a more pronounced effect compared with EMA-based copolymers, likely due to the higher saccharide content. Also in this case, values are consistent with both the type and content of the saccharide monomers. The most pronounced decrease is observed for TP1, likely due to its higher ATR content and, consequently, a greater number of hydroxyl functionalities. TP2 also exhibits a significant reduction in solubility, which can be attributed to the presence of trehalose, offering more hydroxyl groups than the methylglucoside units in TP4.

In all cases, the possible cross-linking can be used to improve the adhesion properties that have been tested on organic solvent-based formulations of some of our polymers. Indeed, CP2, CP6, TP2, and TP4 were selected, as their comonomer ratios closely reflect those of potential industrial interest. The experimental procedure was based on EN 205:2016, the standard method for measuring the tensile shear strength of lap joints for nonstructural thermoplastic wood adhesives (Figure 6). To meet the specific goals of this study, the specimen clamping system was appropriately adapted, as specified in Section 2.5.2, allowing the development of a customized testing protocol. This approach maintained the core principles of the reference standard (namely, the geometry and dimensions of the bonded area, the wood species, and the conditioning of the prepared assemblies prior to testing) while accommodating the particular properties of the synthesized copolymers and terpolymers.

The force–displacement curves are shown in Figure 7 and highlight the mechanical performance of the materials. For each formulation, a representative specimen was selected based on intermediate and significant values of tensile stress, maximum force, and stiffness (Table 7).

**Figure 7:**
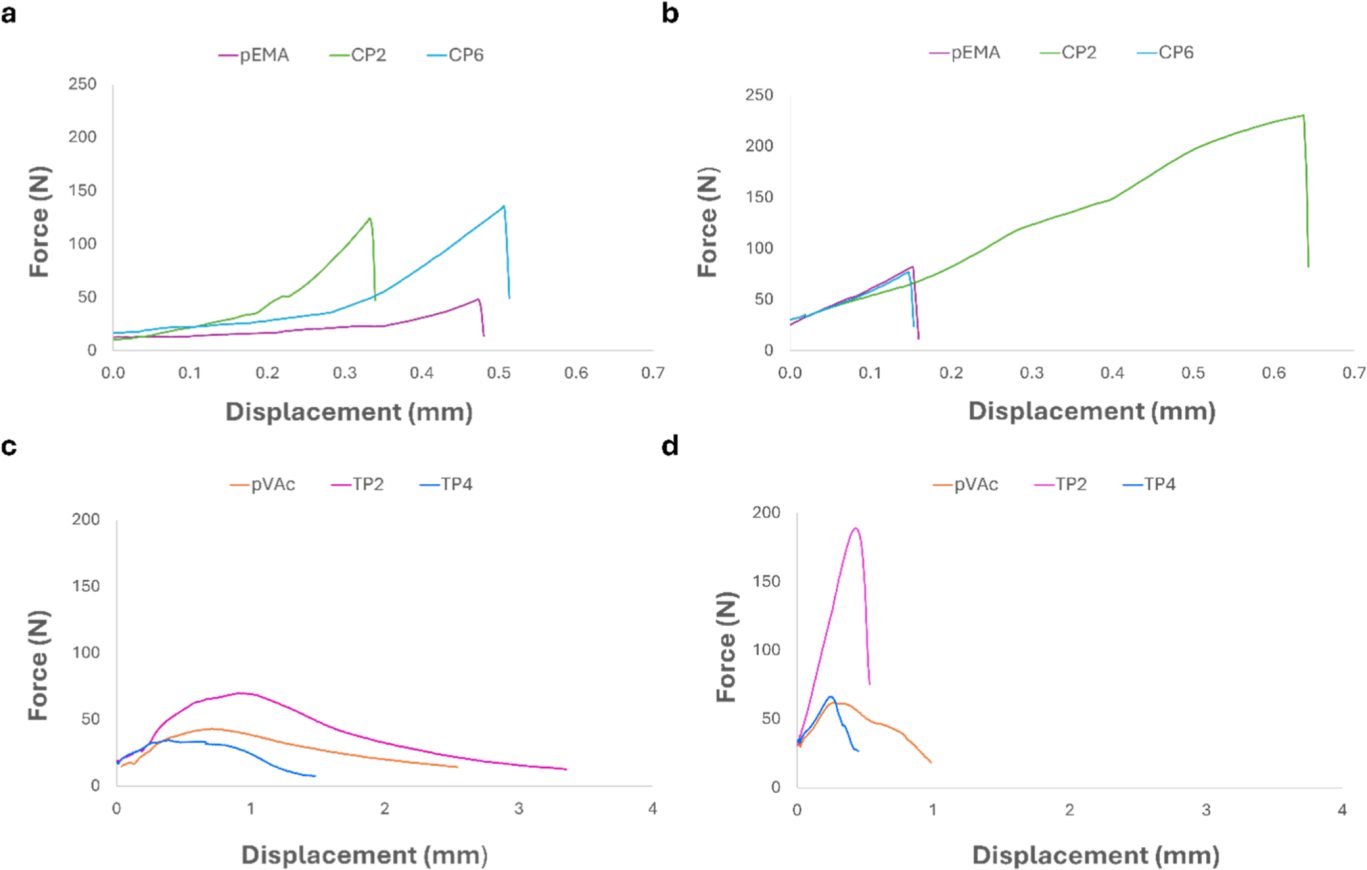
Force–displacement graphs of (a) EMA copolymers, (b) EMA copolymers with isocyanate, (c) VAc/EMA terpolymers, and (d) VAc/EMA terpolymers with isocyanate.

**Table 7:** Tensile Stress, Force at Rupture, and Stiffness Values of Polymers without and with WAT-4^b^

sample		tensile stress (MPa)	force at rupture (N)^a^	stiffness (N/m)
*p*EMA	/	0.4 ± 0.2	75 ± 50	0.3 ± 0.2
	+isocyanate	0.6 ± 0.2	110 ± 47	0.4 ± 0.1
*p*VAc	/	0.2 ± 0.1	43 ± 26	0.1 ± 0.03
	+isocyanate	0.3 ± 0.1	71 ± 18	0.2 ± 0.05
ATR/EMA CP2	/	0.6 ± 0.2	131 ± 47	0.7 ± 0.2
	+isocyanate	0.9 ± 0.4	187 ± 75	0.5 ± 0.2
AMG/EMA CP6	/	0.6 ± 0.1	123 ± 26	0.6 ± 0.2
	+isocyanate	0.5 ± 0.2	110 ± 52	0.5 ± 0.2
ATR/VAc/EMA TP2	/	0.3 ± 0.1	71 ± 24	0.1 ± 0.03
	+isocyanate	0.8 ± 0.2	163 ± 46	0.4 ± 0.2
AMG/VAc/EMA TP4	/	0.1 ± 0.04	17 ± 8	0.1 ± 0.04
	+isocyanate	0.5 ± 0.4	101 ± 82	0.2 ± 0.1

a The force at rupture was recorded directly by the dynamometer at specimen failure. Tensile stress was calculated as the maximum force divided by the cross-sectional area of the bonded interface. Stiffness was determined from the slope of the initial linear region of the force–displacement curve.

b Reported data correspond to seven independent replicates.

Regarding copolymers (Figure 7a), both CP2 and CP6 exhibited greater stiffness than *p*EMA, as indicated by a steeper slope in the force–displacement curve. However, CP6 exhibited higher toughness, defined as the area under the curve, suggesting greater energy absorption before failure. These occurrences indicate that the presence of the copolymer increases interactions between polymer segments of the chain, although these interactions are not strong enough to increase the *T*_g_ (Table 3). Upon isocyanate addition (Figure 7b), the stiffness values became more uniform across samples and CP2 showed a dramatic increase in both toughness and shear strength. These results are consistent with the reactivity tests involving isocyanates, highlighting that the presence of the sugar comonomer (ATR) significantly enhances performance by promoting more effective cross-linking.

In contrast, formulations containing AMG, especially at low concentrations, exhibited behavior comparable to pure EMA. However, although the mechanical characteristics significantly increased with the addition of isocyanate, the brittle response of the joints did not change, and no clear plastic regime was evident in the cured or noncured samples. This implies that the force–displacement curves exhibited elastic behavior up to failure.

The shear strength curves highlighted a pronounced difference between the terpolymers and copolymers. In particular, the terpolymer curves (Figure 7c) showed a more plastic response after the peak force than the copolymer curves, although the postpeak region was in some cases limited. This is evident in the “rounded” shape of the terpolymer curve after rupture; the copolymer curves had a much sharper shape at rupture. This behavior is associated with the low *T*_g_ of *p*VAc and terpolymers (Table 3). Since the test temperature (room temperature) was close to these *T*_g_ values, the polymers exhibited more rubbery characteristics, resulting in the gradual sliding of the adherends prior to failure. This also affected the mechanical properties of the joints, which were generally lower in terpolymers than in copolymers (Table 7). This also applied to the best-performing terpolymer, TP2. For all samples, adding isocyanate (Figure 7d) increased the strength, offsetting the influence of the low *T*_g_. In this case as well, mechanical performance increased most for TP2. TP2 is a combination of VAc, EMA, and ATR; therefore, this outcome was consistent with the reactivity tests with isocyanates, which showed a more pronounced cross-linking effect in the presence of ATR. At the same time, the cross-linking effect, which was particularly evident in TP2, led to narrower plastic behavior at rupture for this polymer, as seen in the force–displacement curves (Figure 7d).

### 3.5 Conclusions

The potential of saccharide-based monomers as functional building blocks for sustainable polymer systems was investigated and confirmed. By introducing allyl-functionalized saccharides (ATR and AMG) into copolymers and terpolymers with EMA and VAc through free radical polymerization, it was possible to tailor polymer reactivity and partially overcome the limitations of low molecular weight reported in previous studies. Appropriate synthetic procedures were developed to optimize the different contributions of the individual monomers in terms of the reactivity. A suitable workup allowed the products to be purified and the main components to be characterized. Spectroscopic analyses confirmed the presence of different monomers, and ^1^H NMR spectroscopy allowed the molar ratio between the different monomer units to be determined. These data, along with gravimetric evaluations, finally allowed the conversions of the different monomers to be monitored. The influence of saccharide monomers on molecular weight and thermal behavior was evaluated by SEC and DSC analysis, comparing the observed results with the number of hydroxyl groups in the saccharide monomer and the ratio of monomeric units in the copolymers. The presence of small quantities of saccharide monomer in the copolymers (1/30, 1/50) and in the terpolymers (1/20/2) allows to maintain MW and *T*_g_ characteristics comparable to the homopolymers of commercial interest (*p*VAc and *p*EMA), while the insertion of monomers with hydroxyl groups can guarantee a better applicative behavior by limiting the presence of emissions of toxic products currently present in some industrial formulations. In fact, the reactivity of the synthesized polymers with isocyanates was confirmed through solubility reduction tests, highlighting the influence of saccharide content and type on cross-linking efficiency. Adhesion tests on beech wood specimens revealed that ATR-containing formulations, especially in the presence of isocyanates, exhibited superior performance compared with AMG-based systems and homopolymers.

Our aim was to investigate the role of the saccharide comonomer, which was systematically evaluated and shown to have a significant influence on the behavior of both acrylic and vinyl polymer chains. However, the assessment of applicative performance can be fully addressed only within the context of a complete adhesive formulation, where additional components and additives may play a crucial role. Therefore, the performances obtained with the solvent-based formulations of the new polymers are not comparable to those obtainable with commercial water-based formulations but represent an important starting point for subsequent studies of industrial formulations.

Overall, these results indicate that saccharide-based copolymers and terpolymers represent promising candidates for the development of partially biobased adhesive systems, combining renewable resources with functional properties suitable for industrial applications.

## Supplementary Material



## Data Availability

No additional data were used for research described in this article.

## Notes

Experimental details; characterization data of monomers and polymers (^1^H and ^13^C NMR, FT-IR, SEC, and DSC); additional NMR spectra; preparation of adhesive formulations; supplementary figure related to adhesive testing; and calculations of saccharide monomer conversion (PDF)

## Glossary

AIBN: azobisisobutyronitrile
AMG: allyl methyl d-glucopyranoside
ATR: allyl α,α′-trehalose
DS: degree of substitution
DSC: differential scanning calorimetry
EMA: ethyl methacrylate
FT-IR: Fourier transform infrared analysis
MEK: methyl methyl ketone
MeOH: methanol
MW: molecular weight
NMR: nuclear magnetic resonance
*p*EMA: poly ethyl methacrylate
*p*VAc: poly vinyl acetate
SEC: size-exclusion chromatography
*T*_g_: glass transition temperature
VAc: vinyl acetate
