# Supplementary material for: Saccharides-Based Polymers for Low Environmental Impact Adhesive Formulations
Source: ACS Omega. 2026 Mar 18;11(12):19010–22. doi: 10.1021/acsomega.5c11562 (PMC13044652; doi:10.1021/acsomega.5c11562)
Supplement: Supplementary file 1 [file ao5c11562_si_001.pdf]

Saccharides-based polymers for low environmental impact adhesive formulations

Alice Cappitti [a*], Emanuele Bianchini [a], Ursula Monaci [a], Daniele Martella [a], Benedetto Pizzo [b], Marco Frediani [a], Antonella Salvini [a*]

[a] University of Florence, Department of Chemistry "Ugo Schiff", Via della Lastruccia 3-13, 50019 Sesto Fiorentino, Italy
[b] CNR-IBE, Istituto per la BioEconomia (Institute of BioEconomy), via Madonna del Piano 10, 50019 Sesto Fiorentino, Italy

* Email: alice.cappitti@unifi.it, antonella.salvini@unifi.it

MONOMERS AND POLYMERS CHARACTERIZATION

Characterization of ATR

^{1}H-NMR (D_2O, 400 MHz, ppm): from 3.32 to 3.88 (m, 12H, H_2–H_6, H_2'–H_6'); from 4.02 to 4.29 (m, 2H, –CH_2–CH=CH_2); 5.13 (m, 2H, H_1, H_1'); from 5.21 to 5.32 (m, 2H, –CH_2–CH=CH_2); 5.89 (m, 1H, –CH_2–CH=CH_2).

Characterization of AMG

^{1}H-NMR (D_2O, 400 MHz, ppm): 3.33 (d, 1H, H_2); 3.35 (s, 3H, –O-CH_3); 3.49 (dd, 1H, H_4), from 3.54 to 3.82 (m, 3H, H_3, H_5, H_6, H_{6b}); from 4.01 to 4.27 (m, 2H, -CH_2-CH=CH_2); 4.73 (m, 1H, H_1^{α}); from 5.18 to 5.31 (m, 2H, -CH_2-CH=CH_2); 5.89 (m, 1H, -CH_2-CH=CH_2).

Characterization of pEMA

^{1}H-NMR (CD_3OD, 400 MHz, ppm): 0.89, 1.06 (s, 3H, -CH_2-C-(CH_3)-); 1.29 (s, 3H, -COO-CH_2-CH_3); 1.91, 1.96 (m, 2H, -CH_2-C-(CH_3)-); 4.06 (m, 2H, -COO-CH_2-CH_3). SEC (Abs. Cal.): M_n = 24810 g/mol; M_w = 38020 g/mol; M_p= 24480 g/mol; Đ = 1.53. DSC (Tg, °C) = 57 (1st cycle); 62 (2nd cycle); 62 (3rd cycle).

Characterization of pVAc

^{1}H-NMR (CD_3OD, 400 MHz, ppm): 1.84 (m, 2H, CH_3-CO-CH-CH_2-); 2.01 (m, 3H, CH_3-CO-CH-CH_2-); 4.98 (m, 1H, CH_3-CO-CH-CH_2-). SEC (Abs. Cal.): M_n = 41730 g/mol; M_w = 73910 g/mol; M_p= 57110 g/mol; Đ = 1.77. DSC (Tg, °C) = 26 (1st cycle); 24 (2nd cycle); 24 (3rd cycle).

Characterization of copolymer EMA/ATR CP1

^{1}H-NMR (CD_3OD, 400 MHz, ppm): 0.89, 1.06 (s, 3H, -CH_2-C-(CH_3)-); 1.29 (s, 3H, -COO-CH_2-CH_3); 1.91, 1.96 (m, 2H, -CH_2-C-(CH_3)-); from 3.44 to 3.95 (m, 10H, H_3-H_6, H_3'-H_6'); 4.06 (m, 2H, -COO-CH_2-CH_3); 4.71 (m, 1H, H_1). SEC (Abs. Cal.): M_n = 20800 g/mol; M_w = 34470 g/mol; M_p= 25010 g/mol; Đ = 1.65. DSC (Tg, °C) = 63 (1st cycle); 67 (2nd cycle); 65 (3rd cycle).

Characterization of copolymer EMA/ATR CP2

^{1}H-NMR (CD_3OD, 400 MHz, ppm): 0.89, 1.06 (s, 3H, -CH_2C(CH_3)-); 1.29 (s, 3H, -COO-CH_2-CH_3); 1.91, 1.96 (m, 2H, -CH_2C(CH_3)-); from 3.44 to 3.95 (m, 10H, H_3-H_6, H_3'-H_6'); 4.06 (m, 2H, -COO-CH_2-CH_3); 4.71 (m, 1H, H_1). SEC (Abs. Cal.): M_n = 21640 g/mol; M_w = 34530 g/mol; M_p= 27820 g/mol; Đ = 1.59. DSC (Tg, °C) = 58 (1st cycle); 64 (2nd cycle); 64 (3rd cycle).

Characterization of copolymer EMA/ATR CP3

^{1}H-NMR (CD_3OD, 400 MHz, ppm): 0.89, 1.06 (s, 3H, -$CH_2C(CH_3)$-); 1.29 (s, 3H, -$COOCH_2CH_3$); 1.91, 1.96 (m, 2H, -$CH_2C(CH_3)$-); from 3.44 to 3.95 (m, 10H, H_3-H_6, H_3'-H_6'); 4.06 (m, 2H, -$COOCH_2CH_3$); 4.71 (m, 1H, H_1). SEC (Abs. Cal.): M_n = 23340 g/mol; M_w = 46870 g/mol; M_p= 29620 g/mol; Đ = 2.01. DSC (Tg, °C) = 54 (2nd cycle).

Characterization of copolymer EMA/AMG CP4

^{1}H-NMR (CD_3OD, 400 MHz, ppm): 0.90, 1.07 (s, 3H, -$CH_2C(CH_3)$-); 1.30 (s, 3H, -$COOCH_2CH_3$); 1.91, 1.96 (m, 2H, -$CH_2C(CH_3)$-); from 3.35 to 3.96 (m, 6H, H_2-H_6); 3.40 (m, 3H, -OCH_3); 4.08 (m, 2H, -$COOCH_2CH_3$); 4.78 (m, 1H, H_1^{α}). DSC (Tg, °C) = 60 (1st cycle).

Characterization of copolymer EMA/AMG CP5

^{1}H-NMR (CD_3OD, 400 MHz, ppm): 0.90, 1.07 (s, 3H, -$CH_2C(CH_3)$-); 1.30 (s, 3H, -$COOCH_2CH_3$); 1.91, 1.96 (m, 2H, -$CH_2C(CH_3)$-); from 3.35 to 3.96 (m, 6H, H_2-H_6); 3.40 (m, 3H, -OCH_3); 4.08 (m, 2H, -$COOCH_2CH_3$); 4.78 (m, 1H, H_1^{α}). SEC (Abs. Cal.): M_n = 46330 g/mol; M_w = 72220 g/mol; M_p= 68310 g/mol; Đ = 1.56. DSC (Tg, °C) = 57 (1st cycle).

Characterization of copolymer EMA/AMG CP6

^{1}H-NMR (CD_3OD, 400 MHz, ppm): 0.90, 1.07 (s, 3H, -$CH_2C(CH_3)$-); 1.30 (s, 3H, -$COOCH_2CH_3$); 1.91, 1.96 (m, 2H, -$CH_2C(CH_3)$-); from 3.35 to 3.96 (m, 6H, H_2-H_6); 3.40 (m, 3H, -OCH_3); 4.08 (m, 2H, -$COOCH_2CH_3$); 4.78 (m, 1H, H_1^{α}). SEC (Abs. Cal.): M_n = 32770 g/mol; M_w = 40800 g/mol; M_p= 34810 g/mol; Đ = 1.24. DSC (Tg, °C) = 61 (1st cycle).

Characterization of terpolymer ATR/VAc/EMA TP1

^{1}H-NMR (CD_3OD, 400 MHz, ppm): 0.90, 1.07 (s, 3H, -CH_2-C-(CH_3)-); 1.27 (s, 3H, -COO-CH_2-CH_3); 1.84 (m, 2H, CH_3-CO-CH-CH_2-); 2.01 (m, 3H, CH_3-CO-CH-CH_2-); from 3.48 to 3.91 (m, 10H, H_3-H_6, H_3'-H_6'); 4.07 (m, 2H, -COO-CH_2-CH_3); 4.98 (m, 1H, CH_3-CO-CH-CH_2-). ^{13}C-NMR (CD_3OD, 400 MHz, ppm): 12.9 (-CH_2-C-(CH_3)-); 19.8 (CH_3-CO-CH-CH_2-); 38.9 (CH_3-CO-CH-CH_2-); 44.5 (-COO-CH_2-CH_3); 60.8 (-COO-CH_2-CH_3); 61.2 (C_6); from 66.2 to 73.3 (C_2-C_6, C_2'-C_6'); 93.6 (C_1); 170.9 (CH_3-CO-CH-CH_2-); 177.3 (-COO-CH_2-CH_3). FT-IR (KBr pellets): 3480 (s, O-H stretching); 2934 (m, C-H stretching); 1739 (s, C═O stretching); 1647 (m, C═C stretching); 1433, 1375 (m, CH_3- bending); 1242 (s, C-O stretching); 1142, 1116, 1085, 1024, (s, C-OH stretching, C-O-C stretching); cm^{-1}. SEC (Abs. Cal.): M_n = 22030 g/mol; M_w = 73790 g/mol; M_p= 14660 g/mol; Đ = 3.35. DSC (Tg, °C) = 35 (1st cycle); 31 (2nd cycle); 31 (3rd cycle).

Characterization of terpolymer ATR/VAc/EMA TP2

^{1}H-NMR (CD_3OD, 400 MHz, ppm): 0.90, 1.07 (s, 3H, -CH_2-C-(CH_3)-); 1.27 (s, 3H, -COO-CH_2-CH_3); 1.84 (m, 2H, CH_3-CO-CH-CH_2-); 2.01 (m, 3H, CH_3-CO-CH-CH_2-); from 3.48 to 3.91 (m, 10H, H_3-H_6, H_3'-H_6'); 4.07 (m, 2H, -COO-CH_2-CH_3); 4.98 (m, 1H, CH_3-CO-CH-CH_2-). ^{13}C-NMR (CD_3OD, 400 MHz, ppm): 12.9 (-CH_2-C-(CH_3)-); 19.8 (CH_3-CO-CH-CH_2-); 38.9 (CH_3-CO-CH-CH_2-); 44.5 (-COO-CH_2-CH_3); 60.8 (-COO-CH_2-CH_3); 61.2 (C_6); from 66.2 to 73.3 (C_2-C_6, C_2'-C_6'); 93.6 (C_1); 170.9 (CH_3-CO-CH-CH_2-); 177.3 (-COO-CH_2-CH_3). FT-IR (KBr pellets): 3480 (s, O-H stretching); 2934 (m, C-H stretching); 1739 (s, C═O stretching); 1647 (m, C═C stretching); 1433, 1375 (m, CH_3- bending); 1242 (s, C-O stretching); 1142, 1116, 1085, 1024, (s, C-OH stretching, C-O-C stretching); cm^{-1}. SEC (Abs. Cal.): M_n = 39710 g/mol; M_w = 69810 g/mol; M_p= 68700 g/mol; Đ = 1.76. DSC (Tg, °C) = 25 (1st cycle); 24 (2nd cycle); 24 (3rd cycle).

Characterization of terpolymer AMG/VAc/EMA TP3

^{1}H-NMR (CD_3OD, 400 MHz, ppm): 0.88, 1.05 (s, 3H, -$CH_2C(CH_3)$-); 1.28 (s, 3H, -$COOCH_2CH_3$); 1.84 (m, 2H, CH_3-CO-CH-CH_2-); 2.04 (m, 3H, CH_3-CO-CH-CH_2-); from 3.34 to 3.96 (m, 6H, H_2^{α}-$H_6{}^{\alpha}$, H_a^{β}-H_6^{β}); 3.40 (m, 3H, -OCH_3) 4.07 (m, 2H, -$COOCH_2CH_3$); 4.66 (m, 1H, H_1^{α}); 4.89 (m, 1H, CH_3-CO-CH-CH_2-). ^{13}C-NMR (CD_3OD, 400 MHz, ppm): 12.8 (-CH_2-C-(CH_3)-); 19.8 (CH_3-CO-CH-CH_2-); 38.8 (CH_3-CO-CH-CH_2-); 44.6 (-COO-CH_2-CH_3); 55.6 (-OCH_3); 60.8 (-COO-CH_2-CH_3); 61.2 (C_6); from 66.2 to 73.8 (C_2-C_6); 99.9 (C_1); 170.9 (CH_3-CO-CH-CH_2-); 177.3 (-COO-CH_2-CH_3). FT-IR (KBr pellets): 3523 (s, O-H stretching); 2981, 2934 (m, C-H stretching); 1739 (s, C=O stretching); 1440, 1375 (m, CH_3- bending); 1242 (s, C-O stretching); 1124, 1024 (s, C-OH stretching, C-O-C stretching); cm^{-1}. SEC (Abs. Cal.): M_n = 33500 g/mol; M_w = 51270 g/mol; M_p= 34900 g/mol; Đ = 1.53. DSC (Tg, °C) = 23 (1st cycle); 23 (2nd cycle); 22 (3rd cycle).

Characterization of terpolymer AMG/VAc/EMA TP4

^{1}H-NMR (CD_3OD, 400 MHz, ppm): 0.88, 1.05 (s, 3H, -$CH_2C(CH_3)$-); 1.28 (s, 3H, -$COOCH_2CH_3$); 1.84 (m, 2H, CH_3-CO-CH-CH_2-); 2.04 (m, 3H, CH_3-CO-CH-CH_2-); from 3.34 to 3.96 (m, 6H, H_2^{α}-$H_6{}^{\alpha}$, H_a^{β}-H_6^{β}); 3.40 (m, 3H, -OCH_3) 4.07 (m, 2H, -$COOCH_2CH_3$); 4.66 (m, 1H, H_1^{α}); 4.89 (m, 1H, CH_3-CO-CH-CH_2-). ^{13}C-NMR (CD_3OD, 400 MHz, ppm): 12.8 (-CH_2-C-(CH_3)-); 19.8 (CH_3-CO-CH-CH_2-); 38.8 (CH_3-CO-CH-CH_2-); 44.6 (-COO-CH_2-CH_3); 55.6 (-OCH_3); 60.8 (-COO-CH_2-CH_3); 61.2 (C_6); from 66.2 to 73.8 (C_2-C_6); 99.9 (C_1); 170.9 (CH_3-CO-CH-CH_2-); 177.3 (-COO-CH_2-CH_3). FT-IR (KBr pellets): 3523 (s, O-H stretching); 2981, 2934 (m, C-H stretching); 1739 (s, C=O stretching); 1440, 1375 (m, CH_3- bending); 1242 (s, C-O stretching); 1124, 1024 (s, C-OH stretching, C-O-C stretching); cm^{-1}. SEC (Abs. Cal.): M_n = 47490 g/mol; M_w = 84380 g/mol; M_p= 59230 g/mol; Đ = 1.78. DSC (Tg, °C) = 24 (1st cycle); 23 (2nd cycle); 23 (3rd cycle).

WOOD SPECIMENS

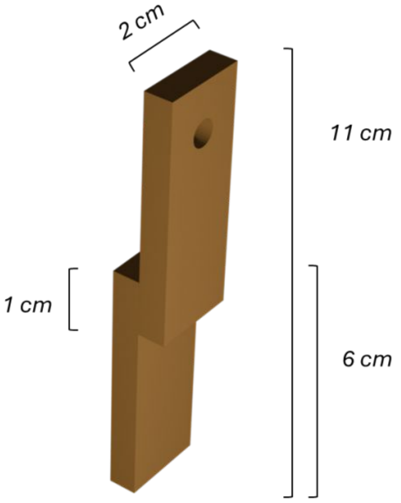

Figure S1. Adhesives tests specimens

SOLUTION PREPARATION FOR ADHESIVE TESTS

Series without isocyanate: ***pEMA***. A 40% (w/w) solution was prepared, containing 500 mg of pEMA and 932 µl of MEK (750 mg). A volume of 47 µl was deposited on each adherend; ***pVAM***: a 40% (w/w) solution was prepared, containing 500 mg of pVAM and 932 µl of MEK (750 mg). A volume of 44.7 µl was deposited on each adherend; ***CP2***: a 40% (w/w) solution was prepared, containing 500

mg of CP1 and 932 µl of MEK (750 mg). A volume of 47 µl was deposited on each adherend; ***CP6***: a 40% (w/w) solution was prepared, containing 500 mg of CP2 and 932 µl of MEK (750 mg). A volume of 42 µl was deposited on each adherend; ***TP2***: a 40% (w/w) solution was prepared, containing 500 mg of TP1 and 932 µl of MEK (750 mg). A volume of 45 µl was deposited on each adherend; ***TP4***: a 40% (w/w) solution was prepared, containing 500 mg of TP2 and 932 µl of MEK (750 mg). A volume of 40 µl was deposited on each adherend.

Series with isocyanate: ***pEMA + isocyanate***: a 40% (w/w) solution was prepared, containing 500 mg of pEMA and 932 µl of MEK (750 mg). 250 mg of Easaqua M502 was added to the solution. A volume of 47 µl was deposited on each adherend; ***pVAM + isocyanate***: a 40% (w/w) solution was prepared, containing 500 mg of pVAM and 932 µl of MEK (750 mg). 250 mg of Easaqua M502 was added to the solution. A volume of 45 µl was deposited on each adherend; ***CP2 + isocyanate***: a 40% (w/w) solution was prepared, containing 500 mg of CP2 and 932 µl of MEK (750 mg). 250 mg of Easaqua M502 was added to the solution. A volume of 47 µl was deposited on each adherend; ***CP6 + isocyanate***: a 40% (w/w) solution was prepared, containing 500 mg of CP6 and 932 µl of MEK (750 mg). 250 mg of Easaqua M502 was added to the solution. A volume of 42 µl was deposited on each adherend; ***TP2 + isocyanate***: a 40% (w/w) solution was prepared, containing 500 mg of TP2 and 932 µl of MEK (750 mg). 250 mg of Easaqua M502 was added to the solution. A volume of 45 µl was deposited on each adherend; ***TP4 + isocyanate***: a 40% (w/w) solution was prepared, containing 500 mg of TP4 and 932 µl of MEK (750 mg). 250 mg of Easaqua M502 was added to the solution. A volume of 40 µl was deposited on each adherend.

^{1}H-NMR SPECTRA

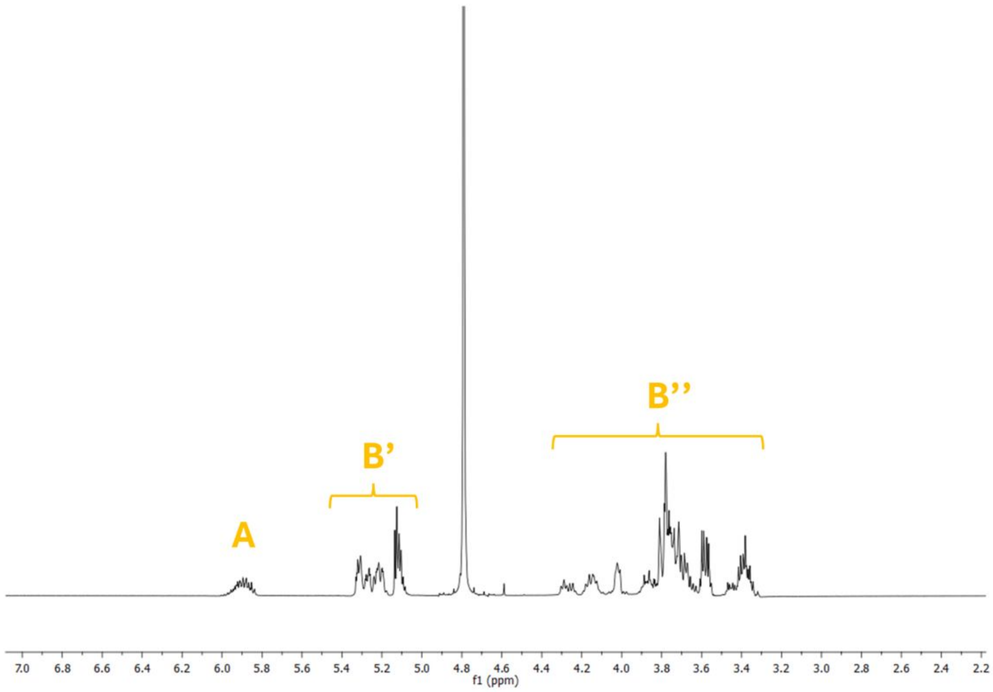

Figure S2. ^{1}H-NMR spectrum of ATR (D_2O)

$$DS\ ATR = \frac{A\ x\ N}{(B'-2A)+(B''-2A)} \qquad \text{(S1)}$$

Where:

- **A** is the value of the integral of the signal that is located at about 5.89 ppm corresponding to the proton of the allyl group ($–CH_2\text{-}C\mathbf{H}=CH_2$);

- **N** is the total number of protons of the disaccharide (14 in the case of α,α'-trehalose);
- **B'** is the value of the integral of the signals at about 5.21-5.32 ppm corresponding to the –CH_2-CH=C$\mathbf{H_2}$ of the allyl group and to the **H_1, H_1'** of α,α'-trehalose;
- **B''** is the value of the integral of the signals at about 3.32-4.29 corresponding to the –C$\mathbf{H_2}$-CH=CH_2 of the allyl group and to the **H_2-H_6**, **H_2'- H_6'** of α,α'-trehalose.

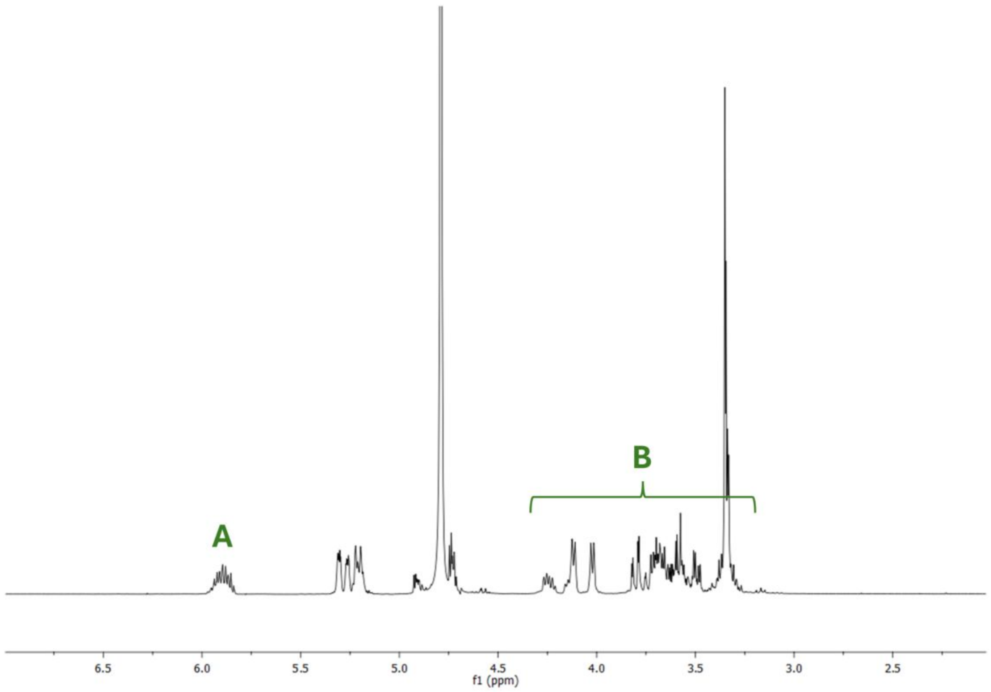

Figure S3. ^{1}H-NMR spectrum of AMG (D_2O)

$$DS\ AMG = \frac{A}{B/9} \qquad (S2)$$

Where:

- **A** is the integral of the signal at 5.89 ppm that corresponds to an H of the allyl group ((-CH_2-C**H**=CH_2));
- **B** is the integral of the signal between 3.34 and 4.35 ppm that integrates 9 H (**H_2**, **H_3**, **H_4**, **H_5**, **H_{6a}**, **H_{6b}**, O**CH_3**);
- **B/9** corresponds to a H of the methyl glucoside.

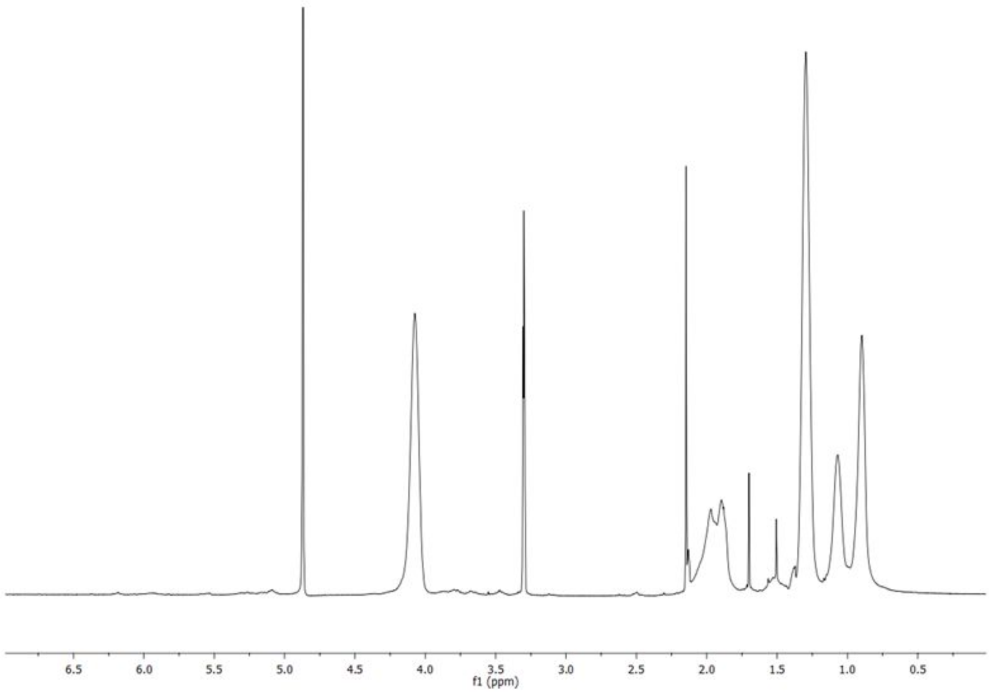

Figure S4. ^{1}H-NMR of CP2 water insoluble fraction (CD_3OD)

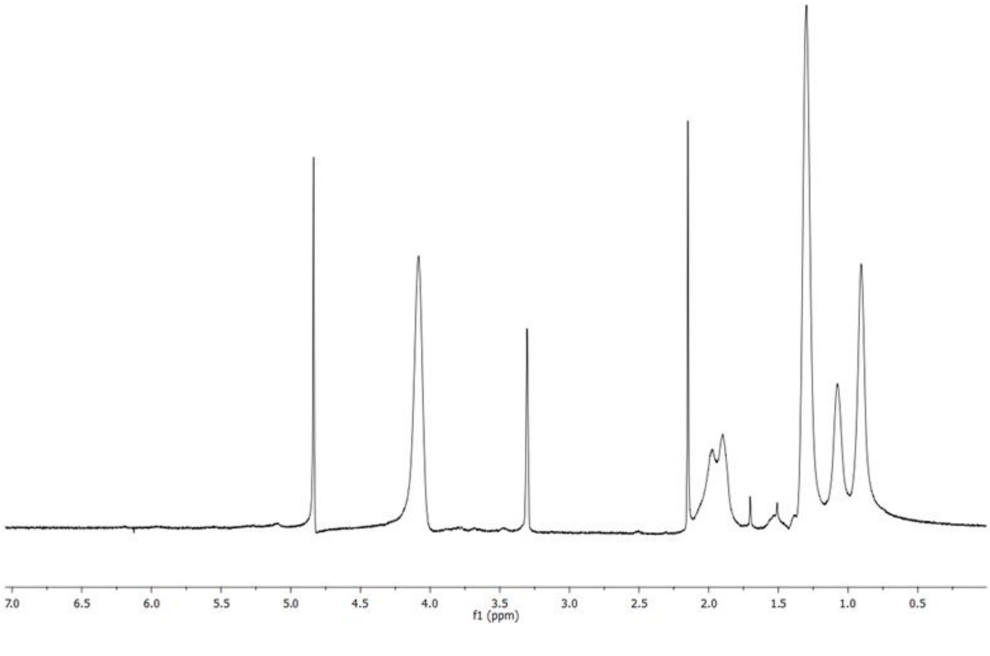

Figure S5. *^{1}H-NMR of CP3 water insoluble fraction (CD_3OD)*

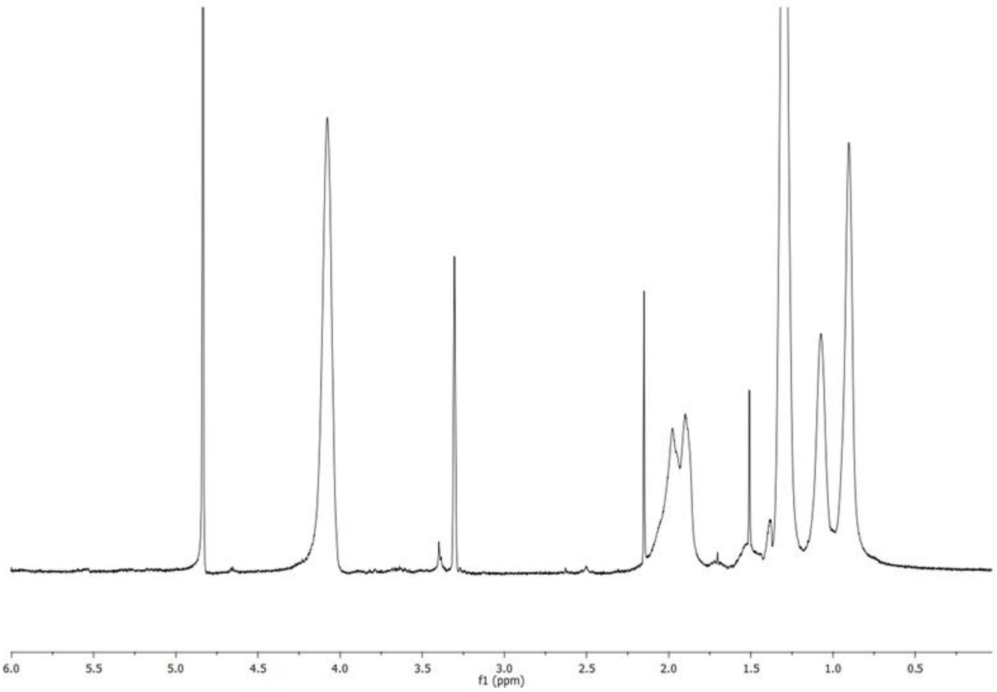

Figure S6. ^{1}H-NMR of CP5 water insoluble fraction (CD_3OD)

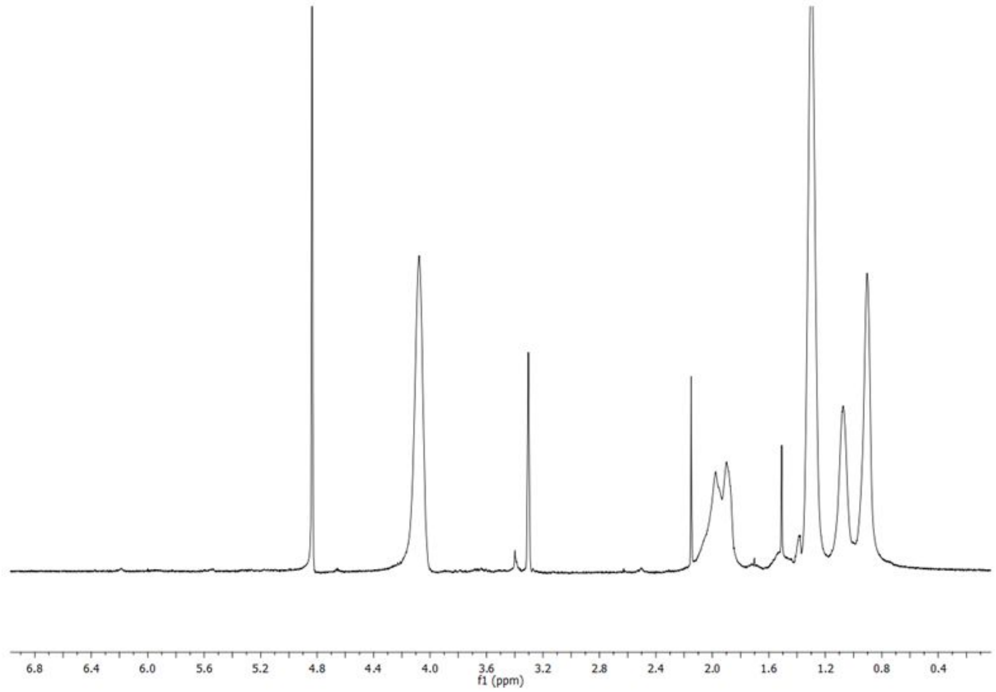

Figure S7. ^{1}H-NMR of CP6 water insoluble fraction (CD_3OD)

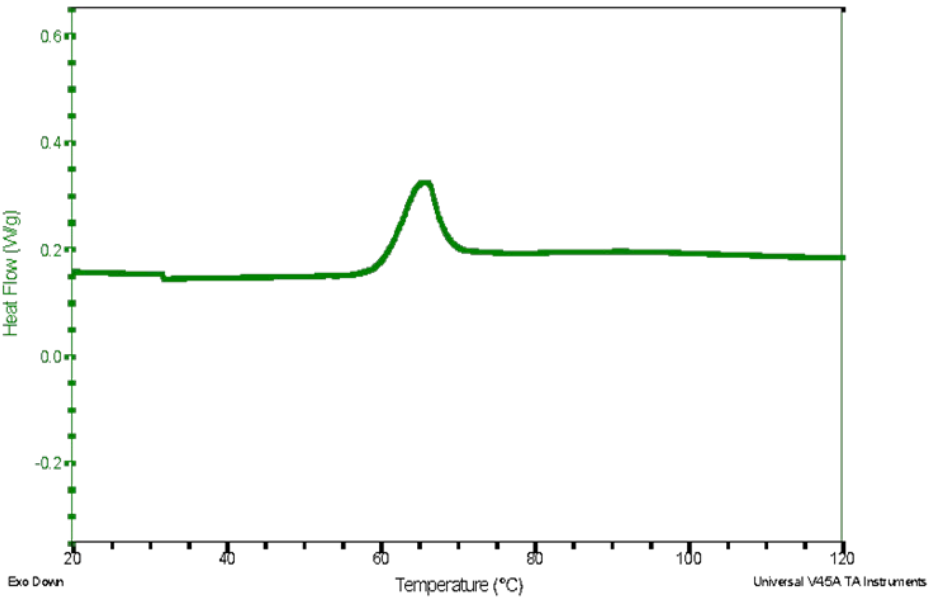

Figure S8. DSC curve of CP3 (1st cycle)

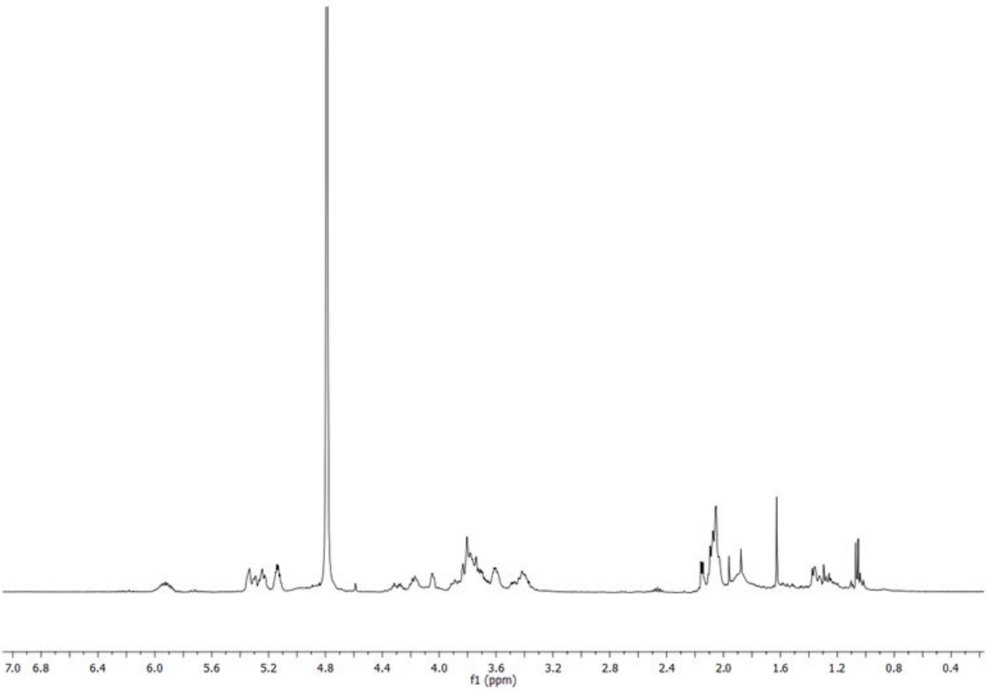

Figure S9. ^{1}H-NMR of TP1 water soluble fraction (CD_3OD)

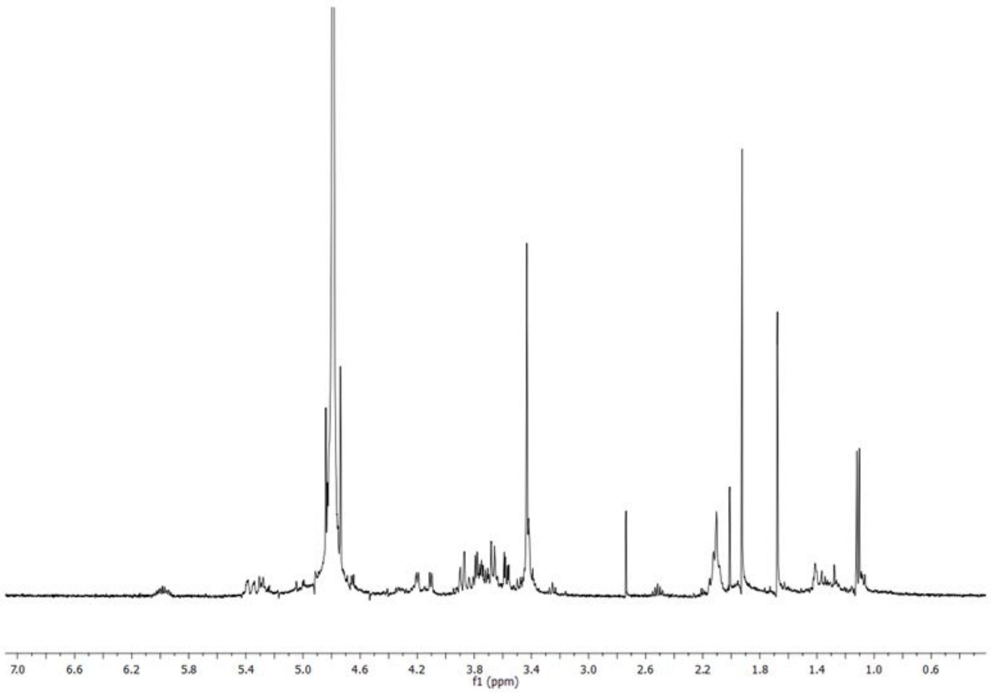

Figure S10. ¹H-NMR of TP3 water soluble fraction (CD_3OD)

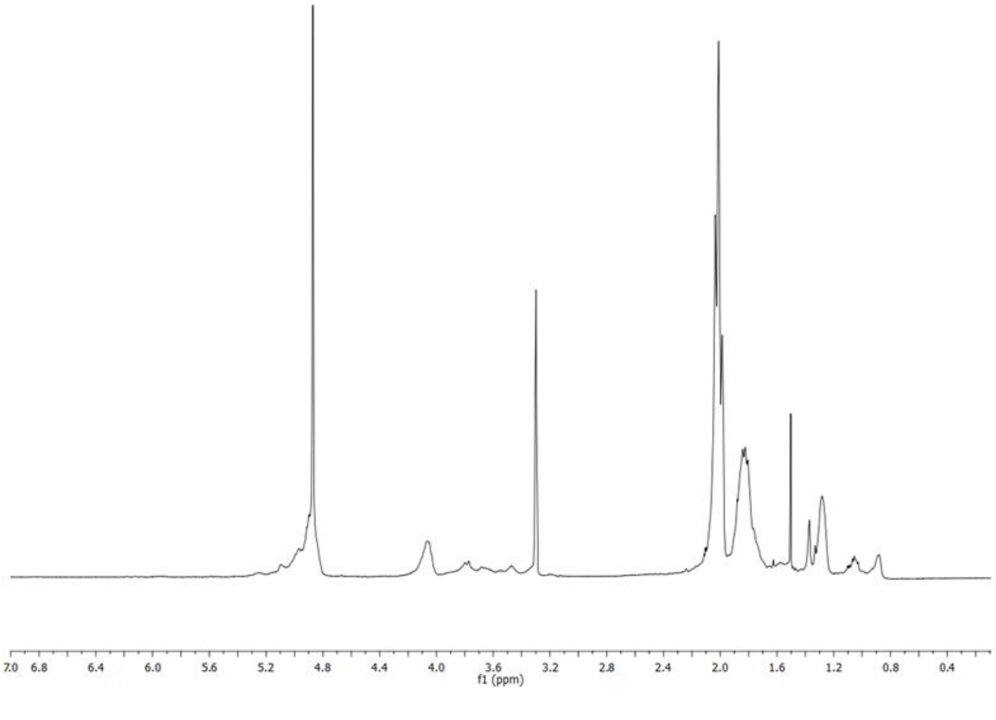

Figure S11. ¹H-NMR of TP2 water insoluble fraction (CD_3OD)

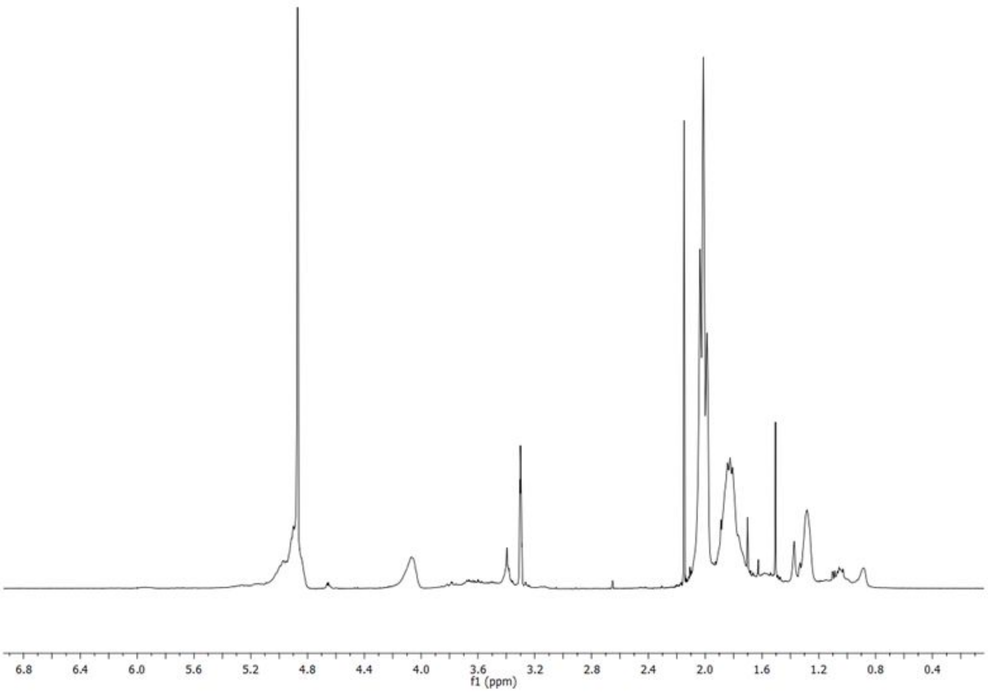

Figure S12. ^{1}H-NMR of TP4 water insoluble fraction (CD_3OD)

Terpolymers: details on the total conversion of saccharide monomers (Figure S13)

Based on the unit ratios (X:Y:Z) and the weight of the water insoluble fraction, it is possible to estimate the percentage of X present in the water insoluble terpolymer fraction, and consequently the percentage present in the water soluble fraction. Finally, considering the change in the degree of substitution (**Figure S9** and **Figure S10** show ^{1}H-NMR of TP1 and TP3 for example) for X in the water soluble extract, it can be calculated the reacted X in that fraction, and consequently total X conversion (**Table S1**).

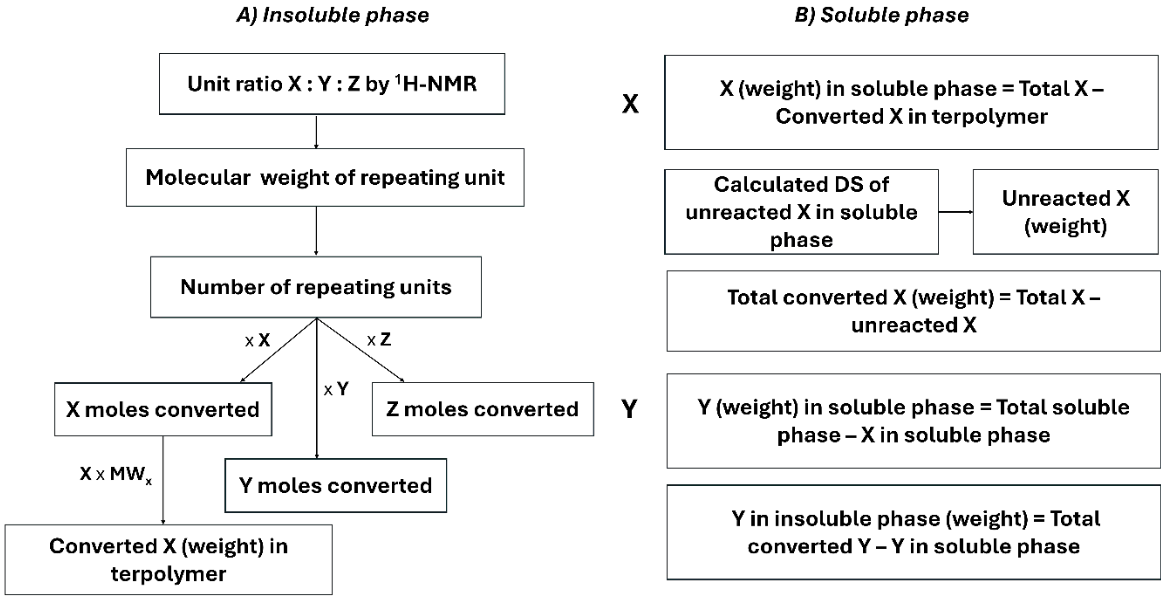

Figure S13. Schematic representation of calculated conversions

Table S1. Further details on monomers conversion

	TP1	TP2	TP3	TP4
**Final crude product (mg)**	247.2	511.2	189.1	351.8
**Water soluble fraction (mg)**	30	26.9	19.3	2.7
**Water insoluble fraction (mg)**	217.4	474.3	148.6	340.7
**ATR/AMG DS in water soluble fraction**	0.58	0.44	0.45	0.27
**Total ATR/AMG conversion (%)**	73.9 (54.9 in the water insoluble copolymer and 18.9 in the water soluble copolymer)	84.8 (55.3 in the water insoluble copolymer and 29.5 in the water soluble copolymer)	72.8 (44 in the water insoluble copolymer and 28.9 in the water soluble copolymer)	99.3 (73.6 in the water insoluble copolymer and 18.8 in the water soluble copolymer)
